# WIDOCK: a reactive docking protocol for virtual screening of covalent inhibitors

**DOI:** 10.1007/s10822-020-00371-5

**Published:** 2021-01-18

**Authors:** Andrea Scarpino, László Petri, Damijan Knez, Tímea Imre, Péter Ábrányi-Balogh, György G. Ferenczy, Stanislav Gobec, György M. Keserű

**Affiliations:** 1 Medicinal Chemistry Research Group, Research Centre for Natural Sciences, Magyar tudósok krt 2, 1117 Budapest, Hungary; 2 Faculty of Pharmacy, University of Ljubljana, Aškerčeva 7, 1000 Ljubljana, Slovenia; 3 MS Metabolomic Research Laboratory, Research Centre for Natural Sciences, Magyar tudósok krt 2, 1117 Budapest, Hungary

**Keywords:** Covalent docking, Virtual screening, Covalent inhibitors, Warhead reactivity

## Abstract

Here we present WIDOCK, a virtual screening protocol that supports the selection of diverse electrophiles as covalent inhibitors by incorporating ligand reactivity towards cysteine residues into AutoDock4. WIDOCK applies the reactive docking method (Backus et al. in Nature 534:570–574, 2016) and extends it into a virtual screening tool by introducing facile experimental or computational parametrization and a ligand focused evaluation scheme together with a retrospective and prospective validation against various therapeutically relevant targets. Parameters accounting for ligand reactivity are derived from experimental reaction kinetic data or alternatively from computed reaction barriers. The performance of this docking protocol was first evaluated by investigating compound series with diverse warhead chemotypes against KRAS^G12C^, MurA and cathepsin B. In addition, WIDOCK was challenged on larger electrophilic libraries screened against OTUB2 and NUDT7. These retrospective analyses showed high sensitivity in retrieving experimental actives, by also leading to superior ROC curves, AUC values and better enrichments than the standard covalent docking tool available in AutoDock4 when compound collections with diverse warheads were investigated. Finally, we applied WIDOCK for the prospective identification of covalent human MAO-A inhibitors acting via a new mechanism by binding to Cys323. The inhibitory activity of several predicted compounds was experimentally confirmed and the labelling of Cys323 was proved by subsequent MS/MS measurements. These findings demonstrate the usefulness of WIDOCK as a warhead-sensitive, covalent virtual screening protocol.

**Supplementary Information:**

The online version contains supplementary material available at 10.1007/s10822-020-00371-5.

## Introduction

Protein ligands with covalent mechanism of action became increasingly popular in both chemical biology and medicinal chemistry applications [1–3]. These compounds form covalent bonds with a targetable nucleophilic residue (most often cysteine, but also others, like lysine, serine, threonine or tyrosine) in an appropriate position at the ligand binding site [4]. Many previous studies have described the advantages and disadvantages of covalent enzyme inhibitors [5–7]. Potential advantages include increased ligand efficiency, prolonged duration of action leading to less frequent dosing, and the opportunity to target shallow binding sites that were previously considered as “undruggable”. Most often cited drawbacks of the covalent mechanism of action are related to their potential of idiosyncratic toxicities that points out the importance of the balanced optimization of their affinity and reactivity.

These compounds interact with the target first by forming a non-covalent protein–ligand complex, then the covalent bond is formed [8]. The functional moiety responsible for the covalent bond formation, also known as the “warhead”, is in most cases an electrophilic reactive group. Many functional groups in organic chemistry are able to react with thiol groups, and they are potential warheads for compounds intended to modify cysteine residues of biological systems. Since different chemotypes bind via different reaction mechanisms, it is clear that they are characterized by distinct intrinsic reactivities. Furthermore, the intrinsic reactivity of the warheads can be tailored by their substituents. Recently we showed [9] that the intrinsic reactivity of the electrophilic ligands might influence not only enzyme specificity, but also functional specificity (as observed with the endo- versus exo-peptidase activity of cathepsin B), and species specificity (as observed for MurA from *Escherichia coli* versus MurA from *Staphylococcus aureus*). These data confirmed that cysteine residues can be labelled by a variety of warheads, which therefore have to be tailored to the reactivity of the specific residue being targeted.

As alternatives to experimental approaches, virtual screening protocols give significant contribution to the identification of viable chemical starting points. However, their application to covalent inhibitors still faces challenges that derive mainly from the description of covalent bond formation. Conventional non-covalent docking methods are designed to well describe the first step, namely the formation of the non-covalent complex. Despite various covalent docking-scoring tools were recently developed, there is no general computational protocol to properly describe the close contact between reacting atoms, the formation of the covalent bond in a chemical reaction, and the conformations and interactions of the resulting complex.

Covalent docking tools follow different strategies to dock and rank covalent binders. For example, GOLD [10–12] uses the post-reaction conformation to rank ligands in a set. The best performing covalent docking protocol developed in AutoDock 4.2 [13] (from now referred to as AD4) is currently the flexible side chain method [14]. This uses post-reaction ligand structures and their conformations are sampled to optimize the interactions in the binding pocket. ICM-Pro [15, 16] generates bound complexes and then ranks ligands poses by excluding the interactions of atoms directly neighboring the newly formed covalent bond. The “Pose Prediction” mode of CovDock [17] combines pre- and post-reaction states. It first performs a non-covalent docking into a binding site where the reactive residue is mutated to Ala in order to avoid close contacts. Next, the rotamer states of the reacting residue are sampled to form the covalent complex with ligand poses occupying beneficial positions according to the previous non-covalent docking step. The final ranking of the ligands is achieved by scoring both pre- and post-reaction states. The “Virtual Screening mode” of CovDock [18] increases the throughput by reducing the number of simulated steps at the expense of somewhat lower binding mode prediction accuracies. A general feature of currently available docking protocols is that they do not explicitly take into account the reaction energy accompanying the covalent bond formation. As a consequence, these docking tools assume that screened ligands have similar intrinsic reactivity that could not be confirmed a priori. Although most of the current covalent docking applications are restricted to a preselected warhead chemotype, intrinsic reactivities are influenced by the substituents at the electrophilic center and should be considerably different.

These limitations of the available covalent docking tools prompted us to develop WIDOCK, a protocol that applies the reactive docking methodology described by Backus et al. [19] and repurposes it into a warhead-sensitive virtual screening solution for diverse electrophilic libraries. The interaction between the ligand and protein atom pairs that are expected to form the covalent bond is modeled by incorporating a pseudo-Lennard–Jones potential into the non-covalent AD4 scoring function. This approach does not involve the formation of a chemical bond between the ligand and the nucleophilic residue, but it rather focuses on the prediction of the non-covalent interactions occurring in the binding pocket before the covalent bond formation and uses a reactivity-scaled reward for compounds able to place the reactive group in the cysteine vicinity. A similar protocol was also described by Forli and Botta [20] to overcome AutoDock’s limitations in treating flexible ring systems. WIDOCK applies the same form of the interatomic potential as in the reactive docking method (see later), with the important difference that we derive the parameters of the potential either from kinetic data measured in reactions of various small compounds against cysteine surrogates, or from calculated quantum chemical reaction barriers. This is a significant simplification with respect to the reactive docking method [19] and its adaptations [21, 22] where parameters were derived from large scale and expensive proteome analysis. Furthermore, while the cited methods were only used to predict cysteines that are most likely to be labeled across the human proteome and to interpret residue ligandability by compounds with limited warhead types, our objective here is to validate and apply WIDOCK as a virtual screening tool. Therefore, we investigate the labeling of reactive cysteine residues of several validated drug targets with compounds having various warheads reacting with diverse chemistries. The main advantage of WIDOCK compared to other available virtual screening tools is that a set of ligands with several warhead types and inherently different cysteine reactivities can be screened against the target of choice and prioritized for experimental testing. In contrast to covalent docking in AD4, this protocol does not require the initial modification of all structures in the set into their post-reaction conformation. While WIDOCK, as a virtual screening tool, primarily aims to discriminate actives from inactives, predicting the ligand conformation in the binding pocket is also a key aspect in the prospective design of covalent inhibitors. In a former study, we showed that non-covalent docking can provide good accuracy in the binding mode prediction of covalent binders, in a reduced time-scale as compared to covalent docking [23]. However, assessing the pose prediction accuracy of WIDOCK would require consistent reactivity data not currently available for a large set of covalent complexes [1, 24].

**Fig. 1:**
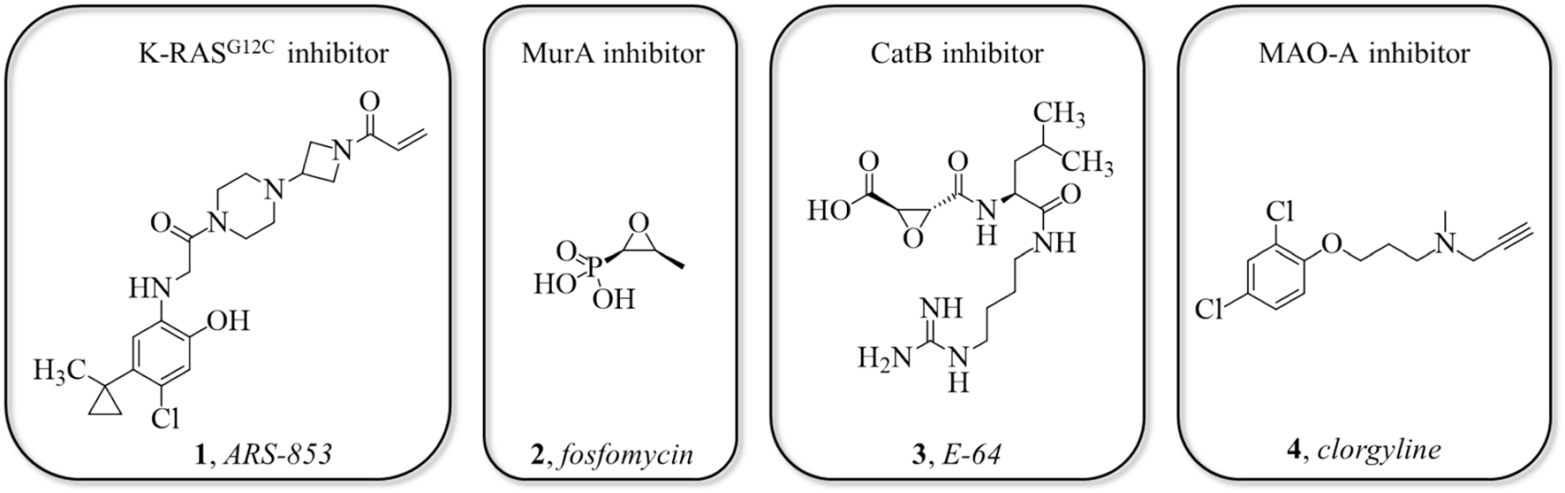
Known covalent inhibitors of the proteins targeted in this study: ARS-853 for KRAS^G12C^, fosfomycin for MurA, E-64 for CatB and clorgyline for MAO-A

In the forthcoming sections we first show that by deriving the warheads’ reactivity parameters for the interaction potential from kinetic measurements against β-mercaptoethanol (BME), WIDOCK accurately reproduces the observed inhibitory activities found against KRAS^G12C^ [25]. KRAS is a widely studied GTPase having multiple oncogenic mutations found in almost 30% of human cancers. The G12C mutation has been shown to reduce the GTP/GDP exchange rate and consequently hyperactivates the enzyme causing abnormal cell growth [26]. KRAS^G12C^ covalent inhibitors targeting Cys12 can selectively bind to the oncogenic variant over the wild-type protein, thus leading to favorable activity modulation [27, 28]. ARS-853 [29] (**1**, Fig. 1), a potent KRAS^G12C^ covalent inhibitor, binds to the GDP-bound oncoprotein thus locking it in its inactive state.

Second, we apply WIDOCK on a covalent fragment library equipped with a diverse set of warheads. This set of compounds was recently screened against MurA (UDP-*N*-acetylglucosamine enolpyruvyl transferase) and cathepsin B (hereinafter also referred to as CatB) [9], and we show that experimental reactivities against glutathione (GSH) can also be used to derive parameters for the pseudo-Lennard–Jones potentials. MurA is a key enzyme in the first step of bacterial peptidoglycan biosynthesis and it is a promising antibacterial target as it has no human orthologue. Despite intense research, relatively few compounds have been described as potent MurA inhibitors [30, 31]. Fosfomycin [32] (**2,** Fig. 1) is the only clinically available MurA inhibitor that binds covalently to the Cys115 residue in the active site. Cathepsin B belongs to the family of lysosomal cysteine proteases and has been validated as a promising therapeutic target in various oncological diseases [33–35]. Although a variety of CatB inhibitors have been developed and investigated for the treatment of different types of cancer, none has yet been approved as a drug [36]. Current covalent inhibitors of CatB are mostly derived from epoxy-succinyl [37], vinyl-sulfone or nitrile warheads [38, 39]. Thus far, one of the most investigated CatB covalent inhibitors is E-64 [40, 41] (**3**, Fig. 1). However, other studies also revealed that different Michael acceptors and halomethyl ketones can be able to form a covalent bond with the active site cysteine [42]. As in the case of KRAS^G12C^, we were able to accurately reproduce experimental screening results for both of the targets.

Furthermore, the diverse set of warheads tested against MurA and CatB allowed us to show that calculated reaction barriers against methyl-thiolate can also be used to parametrize the pseudo-Lennard–Jones potentials. This approach provided comparable results to those obtained by potentials derived from experimental reactivity parameters.

The virtual screening performance of WIDOCK was also evaluated on a larger scale, by screening the electrophilic library compiled and tested by Resnick et al. [43] against OTUB2 and NUDT7. The authors reported thiol reactivity data for the electrophiles in the set, thus allowing us to parametrize WIDOCK accordingly. OTUB2 is a member of the large family of deubiquitinating enzymes (DUBs). OTUB2 is linked to several biological pathways indicating its therapeutic potential for conditions such as viral infections, amyotrophic lateral sclerosis and diabetes [44–46]. NUDT7 is a nudix hydrolase involved in the specific degradation of CoA [47, 48]. Therefore, since CoA metabolism is key for the regulation of glucose homeostasis, also NUDT7 has raised interest for its potential role in the treatment of diabetes [49]. Notably, WIDOCK was able to identify many of the covalent probes identified by Resnick et al. for both these targets, thus showing its scalability in different settings although with varying enrichment rates.

Finally, we have validated WIDOCK prospectively by screening electrophilic fragments against human monoamine oxidase A (MAO-A). MAO-A performs oxidative deamination of monoamine substrates, and plays a key role in the metabolism of neurotransmitters and in the detoxification of amine compounds. Selective inhibitors of MAO-A are used in clinical applications as antidepressants [50–52] as they lead to increased levels of neurotransmitters in noradrenergic and serotoninergic systems [52]. Several irreversible inhibitors of MAO-A have been developed in previous years, such as clorgyline [53] (**4**, Fig. 1). To the best of our knowledge, all reported covalent MAO-A inhibitors with validated labelling position are bound to the FAD cofactor. In contrast, our objective was identifying fragment-sized compounds bound to an active site cysteine and inhibiting the enzyme by blocking the access to the active site. Docking electrophilic fragments to the active site Cys323 of MAO-A by WIDOCK predicted several hits that were confirmed in both biochemical and MS/MS measurements.

Overall, WIDOCK demonstrates that the reactive docking method can be applied as a warhead-sensitive virtual screening tool for the prioritization of covalent binders by incorporating cysteine reactivity parameters into AD4. The usefulness of this approach was demonstrated on a number of retrospective and prospective applications. We believe that the availability of the parameter set and the easy implementation of the protocol into AD4 would facilitate a number of further prospective applications to identify new covalent inhibitors for therapeutic targets.

## Methods

### Compound sets and reactivity data

The electrophilic compounds tested [25] against KRAS^G12C^ (**5**–**24,** structures shown in Supporting Figure S1 and SMILES provided in Supporting Table S1) were evaluated with both standard covalent docking and WIDOCK in order to analyze the predictive power of the two methods against this challenging target. Compound reactivities measured against BME were obtained from [25]. If the reactivity of the particular compound was not explicitly reported, we considered the one of the closest analogue bearing the same electrophilic warhead.

Electrophilic fragments tested against MurA, CatB and MAO-A (**25**–**53**, structures shown in Supporting Figure S2 and SMILES provided in Supporting Table S1) represent eight different warhead chemotypes reacting via two reaction mechanisms: Michael-type nucleophilic addition and nucleophilic substitution. The intrinsic thiol reactivities (see Table 1) were determined by kinetic measurement of adduct formation with *l*-glutathione (GSH), a widely used cysteine surrogate, by an HPLC–MS methodology, as described in the referred study [9].

**Table 1:** Experimental results of the investigated electrophilic fragments in kinetic measurements against l-glutathione (GSH) and in single point enzyme activity assays with MurA and CatB, expressed as residual activities (RA) % at 100 μM

Cmpd	GSH half-life (h)	ln*k*	MurA RA (%)	CatB RA (%)
**25**	7.4	− 2.37	50	106
**26**	20.8	− 3.40	41	97
**27**	48.2	− 4.24	92	79
**28**	41.2	− 4.08	101	87
**29**	127	− 5.21	95	75
**30**	70.1	− 4.62	78	88
**31**	422	− 6.41	80	87
**32**	4.2	− 1.80	51	90
**33**	86.8	− 4.83	59	100
**34**	0.16	1.45	12	87
**35**	94.3	− 4.91	82	77
**36**	4.66	− 1.91	75	70
**37**	16.9	− 3.19	82	84
**38**	< 0.1	1.94	1	91
**39**	< 0.1	1.94	2	87
**40**	< 0.1	1.94	1	89
**41**	< 0.1	1.94	1	82
**42**	161	− 5.45	92	85
**43**	194	− 5.63	75	99
**44**	< 0.1	1.94	0	0
**45**	< 0.1	1.94	2	27
**46**	< 0.1	1.94	3	55
**47**	326	− 6.15	83	86
**48**	32.8	− 3.86	110	88
**49**	5.8	− 2.12	29	106
**50**	95.2	− 4.92	35	91
**51**	22.9	− 3.50	61	87
**52**	8	− 2.45	47	90
**53**	16.1	− 3.15	61	87

For inhibitory activities each data point was acquired twice. SD of the data points was typically less than 10% of the mean. Data taken from [9]

In the larger-scale retrospective virtual screening, an electrophilic fragment library of chloroacetamides and acrylamides was screened against OTUB2 and NUDT7 as described by Resnick and colleagues [43]. In order to have homogeneous reactivity data, we inspected the thiol reactivity reported for the set of 993 compounds and selected those that best fit to the described kinetic model (630 compounds with R^2^ > 0.8 in all three independent thiol screens). As detailed in the referred study, kinetic data were obtained from a high-throughput thiol-reactivity assay measuring the rate of alkylation by the reduced Ellman’s reagent (DTNB, 5,5′-dithio-bis-2-nitrobenzoic acid). The set of 630 electrophiles was then filtered for compounds whose protein labeling was correctly assigned and reported in the cited reference (599 and 616 compounds for OTUB2 and NUDT7, respectively. SMILES provided in Supporting Table S1).

### Ligand preparation

LigPrep [54] from the Schrödinger Suite was used to generate structural isomers, stereoisomers and different protonation states for ligands to be used in both standard and WIDOCK docking simulations. Following the flexible side chain covalent docking protocol available in AD4, ligand structures were modified by attaching the cysteine side chain atoms (Cβ–SG) to the site of alkylation. Modified ligand structures were prepared with LigPrep to generate structural isomers, protonation states and stereoisomers for chiral centers introduced upon cysteine attachment. AutoDockTools was used to prepare structures as PDBQT files for docking calculations.

### Protein preparation

Crystal structures in the Protein Data Bank [55] were used for calculations on KRAS^G12C^, MurA, CatB, OTUB2, NUDT7 and MAO-A. McGregor and colleagues [25] released two structures in the PDB entries 5V6S and 5V6V, in which Cys12 of KRAS^G12C^ is covalently bound to an acrylamide-based (**5**) and an aziridine-based (**7**) inhibitor, respectively. The two structures exhibit significant differences in the conformations of the highly flexible switch II region surrounding the binding site. Therefore, they were both used for docking calculations. For MurA, two protein conformations were considered. For cysteine reactivity predictions, the protein conformation co-crystallized with the cofactor UNAG and the irreversible inhibitor fosfomycin (PDB 1UAE [56]) was evaluated. This is indeed the conformation adopted by the enzyme in the presence of cofactor prior to addition of electrophilic compounds. For docking calculations, the enzyme’s open conformation in the PDB entry 3KQA [57] was preferred over that in 1UAE [56]. This is mainly due to the higher similarity between the fragment library members experimentally validated as MurA actives and terreic acid, the irreversible covalent inhibitor co-crystallized in 3KQA. It suggests that covalent fragments active against MurA are likely to induce a conformational change upon bond formation causing the opening of the loop containing the active site Cys115. A set of molecular descriptors for fosfomycin, terreic acid and MurA actives is provided in Supporting Table S2. For CatB, the recently deposited PDB entry 6AY2 [58] was selected due to its better resolution as compared to other covalent complexes. The residues in the catalytic diad Cys29-His199 were modeled in the thiolate and in the protonated form, respectively. For the retrospective screening against OTUB2 and NUDT7, we selected the high resolution co-crystal structures deposited by Resnick et al. [43] in the PDB entries 5QIV and 5QHA, respectively. For the former, Prime was used to build and optimize the missing side chain of the binding site residue Arg49. The screening was performed by targeting Cys51 in OTUB2 and Cys73 in NUDT7. For the prospective screening against MAO-A, the protein structure was derived from an irreversible complex with clorgyline. Among two available crystal forms, PDB entry 2BXS [59] was preferred over 2BXR [59] as in the latter the targeted cysteine Cys323 is involved in a disulfide bridge with Cys321. Structures were processed by removing co-crystallized inhibitors, water molecules and irrelevant subunits. Protein structures were prepared with the Protein Preparation Wizard in the Schrödinger Suite [60, 61], which was used to add hydrogen atoms, to optimize the H-bond network and to perform a restrained minimization. All targeted cysteines were modelled in the thiolate form, which is acknowledged to be the one participating in the reaction. AutoDockTools was used to prepare structures as PDBQT files for docking calculations.

### Docking calculations

AutoDock4 [13] was used for all docking simulations. Each docking job was defined by a maximum of 100 runs, a population size of 150 individuals, 25 × 10^5^ maximum energy evaluations, 27 × 10^3^ maximum generations and default Lamarckian Genetic Algorithm settings. A grid box of 60 points in each dimension was centered on the targeted cysteine in case of MurA, CatB and MAO-A, whereas for KRAS^G12C^ it was placed on the centroid of the ligand co-crystallized in 5V6S since the same scaffold was present in the set of compounds under investigation. Also for OTUB2 and NUDT7 the grid was centered on the co-crystallized ligands as they both belonged to the screening set. The side chain of the targeted cysteine was modelled as flexible during the non-covalent docking while keeping the rest of the structure rigid. The electrophilic libraries were docked into the target proteins by using the flexible side chain method developed to simulate covalent docking in AD4 [14], the standard non-covalent docking in AD4 and WIDOCK, a reactive docking protocol incorporating ligand reactivity information into the docking simulation. To this end, a new atom type was defined for the electrophilic carbon in the ligand and for the reactive cysteine sulfur with the same set of parameters as the respective standard ones and, following Backus et al. [19] a custom 13–7 pseudo-Lennard–Jones potential was introduced for their interaction. The equilibrium distance (r_eq_) was set to the length of the covalent bond (1.8 Å); kinetic parameters derived from experimental measurements and from calculated activation energies were scaled in a range between 1.0 and 0.175 to model the reactivity of the different ligands and set as the potential well depth (ε_eq_). For each ligand, the best scoring pose was analyzed with a distance-based criterion: if the atoms involved in the formation of the C–S bond were found at a distance lower than 2.20 Å (additional distance is allowed to account for van der Waals repulsions), then the compound was predicted to be a covalent binder. In case of a compound presenting multiple isomers and/or potential reacting centers, all possibilities were enumerated. For each of these, the best scoring pose was analyzed and the one presenting the shortest interatomic distance was considered. Interatomic distances found for all compounds are reported in Supporting Table S3 for the set evaluated against KRAS^G12C^, Supporting Table S4 for the set tested against MurA, CatB and MAO-A, and Supporting Table S5 for the sets screened against OTUB2 and NUDT7. More details on the workflow can be found in the supplementary methods within the Supporting Information.

For covalent docking simulations, the flexible side chain method of AD4 was used. The two additional atoms (Cβ–SG) on ligand structures were used for the alignment on the targeted cysteine in the protein through a SMARTS-based definition of overlapping atoms. After alignment, bound ligands were treated as fully flexible residues during the docking simulation. Compounds were ranked by the score calculated using the semi-empirical force field-based scoring function in AD4. Predicted actives were selected as those retrieved in the top N% of the scoring range, where N varied for each protein target (60% for KRAS, 30% for MurA and MAO-A, 10% for CatB, 7% for OTUB2, 5% for NUDT7) to reflect the fraction of compounds experimentally validated as inhibitors. Covalent docking scores for all compounds in the sets are reported in Supporting Tables S3, S4 and S5. In the KRAS^G12C^ case study, ligands were screened against both protein structures deposited in 5V6S and 5V6V. Following an ensemble approach, WIDOCK and covalent docking poses predicted against the two structures were analyzed. The one with the shorter distance was considered for WIDOCK, and the one with the lower docking score was considered for covalent docking to evaluate virtual screening results.

ROC curves and the area under the ROC curves (AUC) were used as unbiased standard metrics to evaluate the virtual screening performances against all targets. Screening sets were ranked according to C–S interatomic distances and docking scores obtained by WIDOCK and covalent docking, respectively. Furthermore, virtual screening performances were evaluated in terms of the sensitivity, specificity and accuracy displayed by the protocols at the abovementioned target-tailored classification thresholds.

### Cysteine characterization

For electrostatic potential calculations, the quantum mechanical (QM) region was defined by including residues within 4 Å from the targeted cysteine. Single point DFT B3LYP calculations with 6-31G* basis were performed in continuum solvation models and the electrostatic potential on the van der Waals surface of atoms included in the QM region was calculated by using QSite [62–64]. The web-based platform Cpipe [65] was used to predict pK_a_ and cysteine reactivity information. Solvent accessible surface area (SASA) calculations were performed on prepared protein structures by using the POPS algorithm [66].

### QM calculations

DFT methods were used to calculate reaction energies (**ΔG**_***r***_) and activation energy barriers (**ΔG**^**‡**^) against the methyl-thiolate anion (MeS^−^) as a cysteine surrogate. They both have been already validated in predicting reactivities [67–69], however, these studies focused on limited warhead chemotypes. We used the Gaussian 09 software package [70] with the SMD implicit solvation model (water) and the M062X functional [71], as it has been shown as one of the most accurate functional to calculate these parameters [72, 73]. We performed geometry optimizations and estimated the entropic contributions with the 6-311G+(d,p) basis set to obtain both energies (**E**) and Gibbs free energies (**G**). Then we calculated single point energies (**E**′) with the larger basis set 6-311++G(3df,3pd). From these data we calculated the Gibbs free energies (**G**′) of the investigated structures (Eq. 1). We optimized the transition state and product geometries (in the case of Michael acceptors we always considered the s-*cis* transitional geometry [68]) with the 6-311G+(d,p) basis set. QST3 transition state optimization was applied and IRC calculations were performed in order to prove that the transition states connect two corresponding minima. Frequencies were calculated to assure that transition states are on saddle points having one imaginary frequency, and reactants and products are in local minima having no imaginary frequency. Entropic and thermal corrections were evaluated for isolated molecules using standard rigid rotor harmonic oscillator approximations (i.e. Gibbs free energies were taken as the sum of electronic and thermal free energies of vibrational frequency calculations). The H, G and S values were obtained at standard conditions. In addition, single point energies were calculated with the 6-311++G(3df,3pd) basis set. The activation energy barriers (**ΔG**^**‡**^) (Eq. 2) were determined as Gibbs free energy differences of the optimized transition states (${\bf{G}}_{\bf{T}\bf{S}}^{{^{\prime}}}$) and the initial compounds. Analogously, the reaction energies (**ΔG**_***r***_) (Eq. 3) were obtained as the free energy differences of the optimized products (${\bf{G}}_{\text{product}}^{{^{\prime}}}$) and the initial compounds (data are shown in Supporting Table S6).

1 $${\mathbf{G^{\prime}}} = {\mathbf{E^{\prime}}} + \left( {{\mathbf{G}} - {\mathbf{E}}} \right)$$

2 $$\Delta {\mathbf{G}}^{\ddag } = {\mathbf{G^{\prime}}}_{{{\mathbf{TS}}}} - \left( {{\mathbf{G^{\prime}}}_{0} + {\mathbf{G^{\prime}}}_{{{\mathbf{SH}}}} } \right)$$

3 $$\Delta {\mathbf{G}}_{r} = {\mathbf{G^{\prime}}}_{{{\text{product}}}} - \left( {{\mathbf{G^{\prime}}}_{0} + {\mathbf{G^{\prime}}}_{{{\mathbf{SH}}}} } \right)$$

### Inhibitory activity data

Inhibitory activities for compounds **5**–**24** expressed as % of KRAS^G12C^ labeling at 100 μM compound concentration were obtained from [25]. Compounds showing > 50% labeling were considered as actives. Inhibitory activities for compounds **25**–**53** expressed as residual activity of MurA and CatB at 100 μM compound concentration were taken from [9]. Compounds showing < 60% residual activity were considered as actives. Inhibitory activities expressed as % labeling for the virtual screening at 200 μM compound concentration against OTUB2 and NUDT7 were taken from [43]. Compounds showing > 50% labeling were considered as actives.

### MAO-A activity assay

The effects of the test compounds on MAO-A were investigated using a fluorimetric assay, following a previously described methodology [74]. The inhibitory activity of the compounds was evaluated by their effects on the production of hydrogen peroxide (H_2_O_2_) from *p*-tyramine. The production of H_2_O_2_ was detected using Amplex Red reagent in the presence of horseradish peroxidase, where a highly sensitive fluorescent product, resorufin, is produced at stoichiometric amounts. Recombinant human microsomal MAO-A enzyme expressed in baculovirus infected insect cells (BTI-TN-5B1-4), horse-radish peroxidase (type II, lyophilized powder), and *p*-tyramine hydrochloride were obtained from Sigma Aldrich. 10-Acetyl-3,7-dihydroxyphenoxazine (Amplex Red reagent) was synthesized as described in the literature [75].

Briefly, 100 µL 50 mM sodium phosphate buffer (pH 7.4, 0.05% Triton X-114) containing the compounds and MAO-A were incubated for 30 min at 37 °C in a flat-bottomed black 96-well microplate, and placed in a dark microplate reader chamber. After the pre-incubation, the reaction was started by adding the final concentrations of 200 µM Amplex Red reagent, 2 U/mL horseradish peroxidase, and 1 mM *p*-tyramine (final volume, 200 µL). The production of resorufin was quantified based on the fluorescence generated (λ_ex_ = 530 nm, λ_em_ = 590 nm) at 37 °C over a period of 30 min, during which time the fluorescence increased linearly. For control experiments, DMSO was used instead of the appropriate dilutions of the compounds in DMSO. To determine the blank value (b), phosphate-buffered solution replaced the enzyme solution. The initial velocities were calculated from the trends obtained, with each measurement carried out in duplicate. The specific fluorescence emission to obtain the final result was calculated after subtraction of the blank activity (b). The inhibitory potencies are expressed as the residual activities (RA): ${\text{RA}} = \frac{{{\text{v}}_{{\text{i}}} - {\text{b}}}}{{{\text{v}}_{0} - {\text{b}}}},$ where v_i_ is the velocity in the presence of the test compounds, and v_0_ the control velocity in the presence of 1.5% DMSO.

### Labelling of human MAO-A

MAO-A (150 μL, 52 µM) stored in 50 mM Hepes, pH = 7.5 with 0.25% Triton X-100 was thawed at 37 °C, and then desalted using a G-25 (fine) Sephadex column to 50 mM K_3_PO_4_, pH = 7.5 containing 0.25% Triton X-100. The resulted 40 µM sample was divided, 35 µL was taken, and the electrophilic fragment (0.5 µL, 100 mM in DMSO) was added. The mixture was incubated at 4 °C for 24 h.

### Digestion and LC–MS/MS analysis of labelled human MAO-A

The tryptic digestion method was adapted from our former publication [9]. Briefly, 35 μL of MAO-A (40 μM), 10 μL 0.2% (w/v) RapiGest SF (Waters, Milford, USA) solution buffered with 50 mM ammonium bicarbonate (NH_4_HCO_3_) were mixed (pH = 7.8). 3.3 μL of 45 mM DTT (~150 nmol) in 100 mM NH_4_HCO_3_ were added and kept at 37.5 °C for 30 min. After cooling the sample to room temperature, 4.16 μL of 100 mM iodoacetamide (416 nmol) in 100 mM NH_4_HCO_3_ were added and placed in the dark at room temperature for 30 min. The reduced and alkylated protein was then digested by 10 μL (1 mg/mL) trypsin (the enzyme-to-protein ratio was 1:10) (Sigma, St. Louis, MO, USA). The sample was incubated at 37 °C for overnight. To degrade the surfactant, 7 μL of formic acid (500 mM) solution was added to the digested MAO-A sample to obtain the final 40 mM (pH ≈ 2) and was incubated at 37 °C for 45 min. For LC–MS analysis, the acid treated sample was centrifuged for 5 min at 13 000 rpm.

QTRAP 6500 triple quadruple—linear ion trap mass spectrometer, equipped with a Turbo V source in electrospray mode (AB Sciex, CA, USA) and an Agilent 1100 Binary Pump HPLC system (Agilent Technologies, Waldbronn, Germany) consisting of an autosampler was used for LC–MS/MS analysis. Data acquisition and processing were performed using Analyst software version 1.6.2 (AB Sciex Instruments, CA, USA). Chromatographic separation was achieved by using the Discovery® BIO Wide Pore C-18-5 (250 mm × 2.1 mm, 5 μm). The sample was eluted with a gradient of solvent A (0.1% formic acid in water) and solvent B (0.1% formic acid in ACN). The flow rate was set to 0.2 mL/min. The initial conditions for separation were 5% B for 7 min, followed by a linear gradient to 90% B by 53 min, from 60 to 63 min 90% B is retained; from 64 to 65 min back to the initial conditions with 5% eluent B retained to 75 min. The injection volume was 10 μL (300 pmol on the column).

Information Dependent Acquisition (IDA) LC–MS/MS experiment was used to identify the modified tryptic MAO-A peptide fragments. Enhanced MS scan (EMS) was applied as survey scan and enhanced product ion (EPI) was the dependent scan. The collision energy in EPI experiments was set to rolling collision energy mode, where the actual value was set on the basis of the mass and charge state of the selected ion. Further IDA criteria: ions greater than 400,000 m/z, which exceeds 10^6^ counts, exclude former target ions for 30 s after two occurrences. In EMS and in EPI mode the scan rate was 1000 Da/s as well. Nitrogen was used as the nebulizer gas (GS1), heater gas (GS2), and curtain gas with the optimum values set at 50, 40 and 40 (arbitrary units). The source temperature was 350 °C and the ion spray voltage set at 5000 V. Declustering potential value was set to 150 V. GPMAW 4.2 software was used to analyze the large number of MS–MS spectra and identify the modified tryptic MAO-A peptides.

## Results and discussion

### Retrospective docking on KRAS^G12C^

**Fig. 2:**
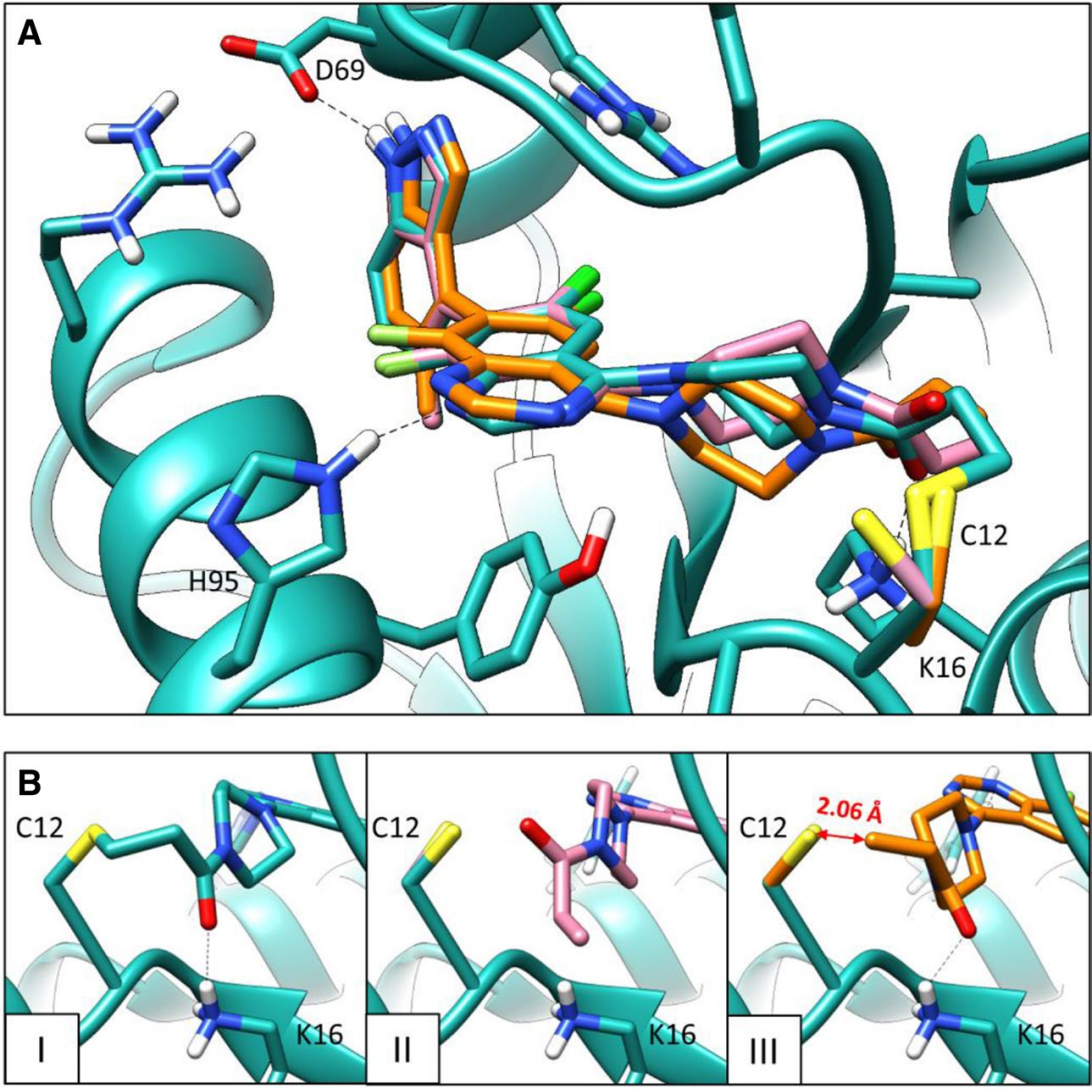
Self-docking of the acrylamide-based KRAS^G12C^ inhibitor **5** co-crystallized in 5V6S. Crystal structure in cyan; AD4 non-covalent docking pose in pink; pose generated by WIDOCK in orange. **a** Overall consensus found in the predicted binding modes with respect to the co-crystallized conformation. **b** Structural differences in the warhead region: *I* warhead conformation in the crystal structure; *II* warhead conformation in the standard non-covalent AD4 pose; *III* warhead conformation in WIDOCK pose

First, WIDOCK was challenged by a set of compounds covalently labeling Cys12 in KRAS^G12C^ with a range of electrophilic warheads. McGregor et al. elaborated a reported KRAS^G12C^ switch II inhibitor scaffold by introducing different warhead types probing Cys12 reactivity. The tested electrophiles included acrylamides, epoxides, aziridines, α-chloroacetamides, β-chloroethylureas, acyl-imidazoles, diazoacetamides and other warheads reacting through nucleophilic substitution. The compounds differ only in the warhead, therefore differences in the inhibitory potency can be assigned to differences in the intrinsic reactivities and in the optimal orientations of the reacting groups. Therefore, this set is perfectly suited to test the docking performance of WIDOCK on a range of warheads. Indeed, the main advantage provided by our method lies in the opportunity to screen and compare covalent ligands bearing multiple warhead types characterized by different intrinsic reactivities. Moreover, the authors assessed the thiol reactivity of the compounds by measuring the extent of covalent adduct formation with β-mercaptoethanol (BME) as a thiol surrogate. Thus, we could use the reported reactivity information to parametrize the customized pseudo-Lennard–Jones potentials for all the compounds. By docking this set of compounds to KRAS^G12C^ we could inspect the ability of WIDOCK to induce a conformational change in the warhead with respect to docking poses generated by the standard non-covalent docking in AD4. As an illustrative example, Fig. 2 shows the binding modes generated for compound **5**, a co-crystallized KRAS^G12C^ covalent inhibitor. Both standard docking and WIDOCK could provide an overall good consensus with the experimentally determined binding mode (Fig. 2a). However, by focusing on the warhead conformation (Fig. 2b), the best scoring standard docking pose predicted an incorrect geometry of the acrylamide moiety (Fig. 2b-I vs. Fig. 2b-II). On the other hand, the reactivity-scaled interaction potential introduced between reacting atoms in WIDOCK induced a flip in the warhead structure, by placing the reactive β-carbon in close proximity to the targeted cysteine sulfur (2.06 Å) (Fig. 2b-III). This result highlights the capability of WIDOCK to predict the correct geometry of the ligand warhead while keeping the correct binding mode in the pocket.

**Fig. 3:**
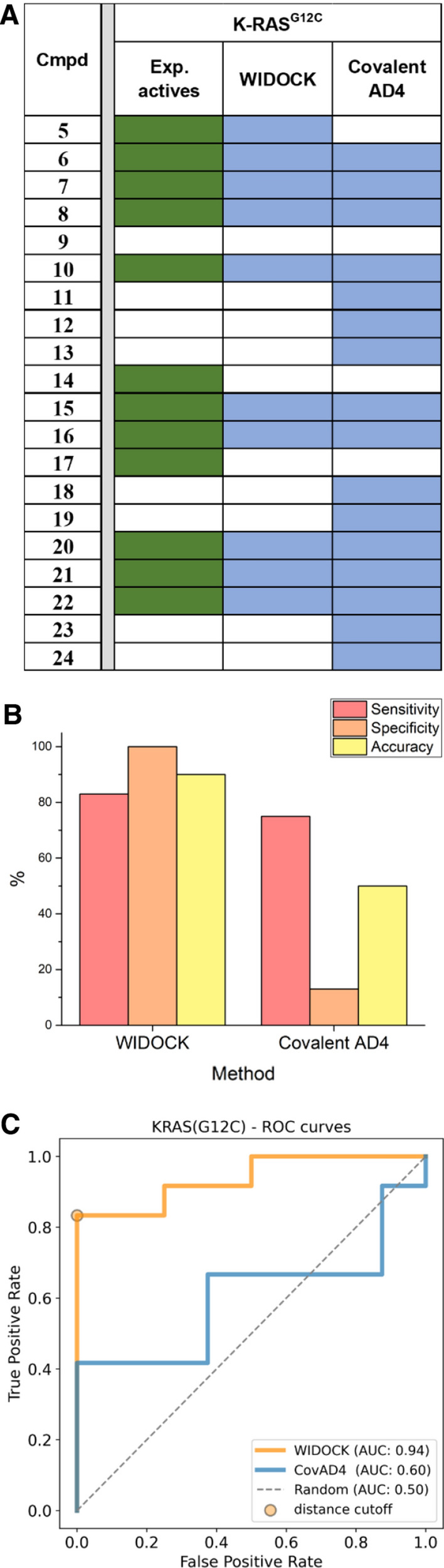
Evaluation of WIDOCK and covalent docking in AD4 (CovAD4) against KRAS^G12C^. **a** Docking results: colored cells represent experimental and predicted actives (in green and blue, respectively). Experimental actives were considered as those showing > 50% KRAS^G12C^ labeling at pH 7.5 at 100 μM concentration. Pseudo-Lennard–Jones potentials for WIDOCK were derived using experimental reactivities against BME. **b** Performance metrics at custom cutoffs. **c** ROC curves

Overall, by using WIDOCK on KRAS^G12C^ we could retrieve 10 out of the 12 experimental actives (except for the acyl-imidazole **14** and the α-chloro-acetamide **17**) within the defined distance cutoff, that represents 83% sensitivity (or True Positive Rate, TPR) (Fig. 3). In comparison, we screened the library using the dedicated covalent docking module of AD4 (flexible side chain method). By analyzing the results based on a target-tailored classification threshold (see “Docking calculations” in “Methods” section), covalent docking provided a TPR of 75% (Fig. 3b), thus somewhat lower than the one achieved by WIDOCK (more details in Supporting Table S3). In addition, covalent docking showed significantly lower specificity and accuracy (13 and 50%) as compared to WIDOCK (100 and 90%), which emphasize the ability of WIDOCK to discriminate active from inactive compounds in the screening set. Additionally, ROC curves were produced as unbiased performance metrics to evaluate virtual screening results (Fig. 3c). The superior performance of WIDOCK relative to covalent AD4 is shown by both the higher early enrichment of actives (Supporting Figure S4) and the larger AUC values, thus supporting the utility of WIDOCK in handling screening sets composed of diverse warhead chemotypes.

### Retrospective docking on MurA and CatB

WIDOCK was next evaluated on electrophilic fragments equipped with diverse warheads against MurA and CatB. In Table 1, we report reactivity and inhibitory activity data for the library members. Their reactivities against *l*-glutathione (GSH) are characterized by the half-life of adduct formation and by the pseudo first order reaction rate constant (*k*) derived from the former. These fragments were measured in single point enzyme activity assays at 100 μM concentration against MurA and CatB. Reactivity and inhibition data were taken from a recent publication [9].

Biochemical results shown in Table 1 suggest that a large fraction of highly reactive compounds led to a better inhibitory profile (lower remaining activity) when tested against MurA. This is in line with the idea that a more pronounced reactivity improves the chance of covalent binding, and consequently enhances the inhibitory activity. This trend, however, is not seen by the results obtained against CatB. This highlights that reactivity is not the only factor driving the binding of covalent ligands, and significant degree of target selectivity can be achieved [43]. The role of the binding site residues involved in the initial ligand recognition, and by the cysteine surroundings affecting its intrinsic nucleophilicity can be interpreted by characterizing the active site cysteines of MurA (Cys115) and CatB (Cys29). We used three tools to obtain reactivity and accessibility descriptors (Table 2). QSite [62–64] by Schrödinger was used to perform mixed QM/MM calculations to inspect the reactive center in the protein. It provided information about the electrostatic potential minima (ESP_min_) on the sulfur atom of the cysteines, thus indicating the relative nucleophilicity of the targeted residues. By using the POPS algorithm, we could retrieve information on the cysteine accessibilities, in terms of solvent accessible surface areas (SASA) of both the whole residue and its side chain sulfur (SG). Finally, the web-based platform Cpipe was used for reactivity and pK_a_ predictions for the analyzed cysteines.

**Table 2:** Parameters indicating reactivity and accessibility of cysteines in MurA and CatB

Target	Residue	QSite	POPS		Cpipe	
		ESP_min_ (kcal/mol)	Cys (whole) SASA (A^2^)	Cys (SG) SASA (A^2^)	pK_a_	Prediction
MurA	C115	− 370.85	34.78	15.1	4.26	Reactive
CatB	C29	− 383.32	19.97	10.7	2.45	Reactive

By investigating the calculated properties, the lower ESP_min_ and pK_a_ values of CatB’s Cys29 compared to MurA’s Cys115 suggest a more pronounced nucleophilicity of the former, which is accompanied, however, by a lower accessibility. Overall, the lower accessibility of the catalytic cysteine in CatB could provide an explanation of the lower number of experimental actives found against this target.

**Fig. 4:**
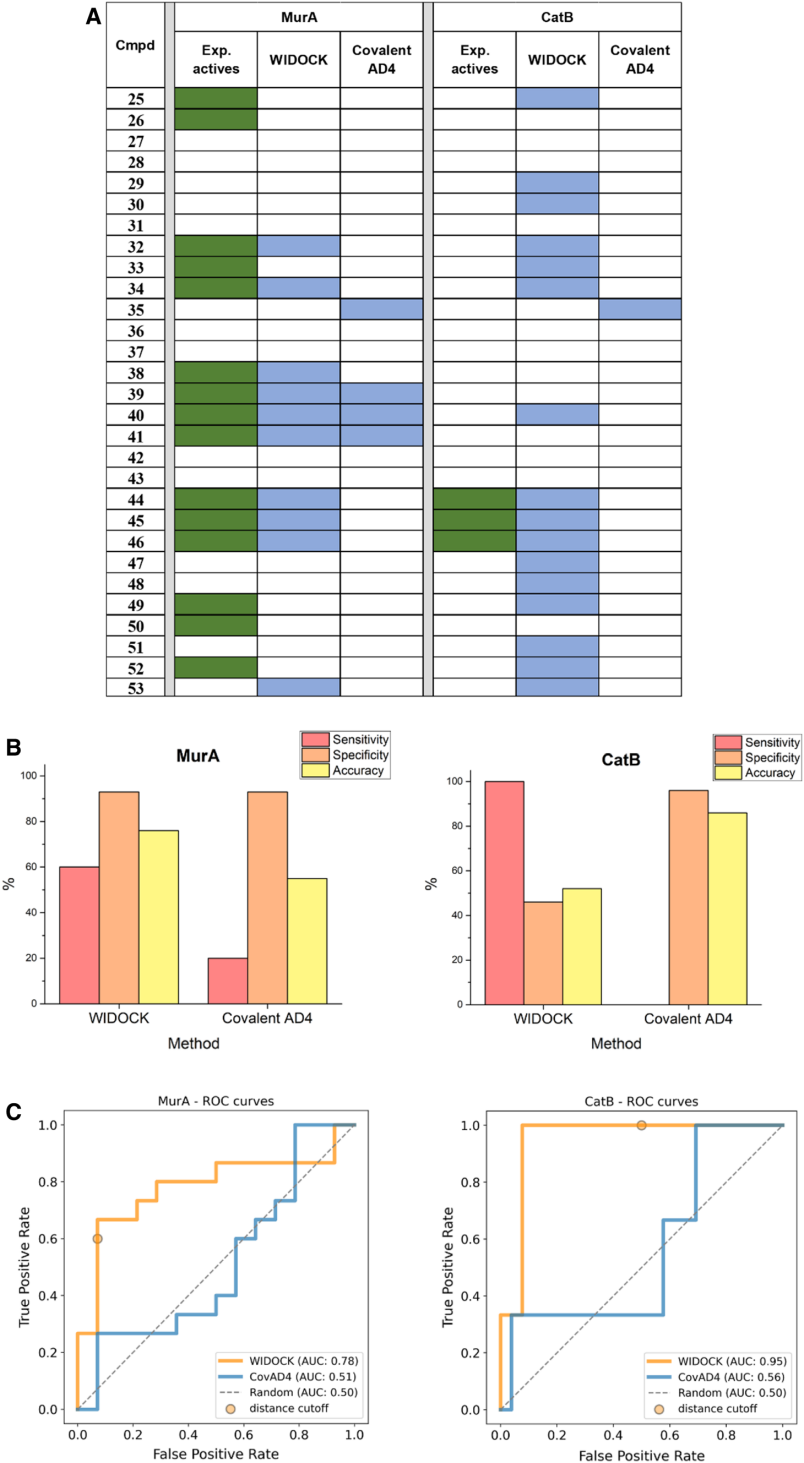
Evaluation of WIDOCK and covalent docking in AD4 against MurA and CatB. **a** Docking results: colored cells represent experimental and predicted actives (in green and blue, respectively). Pseudo-Lennard–Jones potentials were parametrized using experimental reactivities against GSH. **b** Performance metrics at custom cutoffs. **c** ROC curves

The experimental reactivity data measured against GSH (ln*k* in Table 1) were used to derive parameters for WIDOCK. Then, ligands of Table 1 were docked against MurA and CatB by targeting the reactive cysteine being able to modulate the functional activity of each protein when covalently modified. Activity prediction was compared to experimental screening results available for the two enzymes. WIDOCK was able to retrieve most of the validated actives, with few false positives having the reacting atom pair within the defined distance cutoff (Fig. 4a, for detailed dataset see Supporting Table S4). Overall, screening by WIDOCK resulted in 60% and 100% sensitivity against MurA and CatB, respectively (Fig. 4b). The varying performance in terms of true positive rates reflects the different structural framework defined by the binding site residues involved in the non-covalent recognition of covalent ligands. Indeed, the non-covalent interactions formed in the pocket are important to place the warhead in the right position and orientation with respect to the protein nucleophile in order to allow for the chemical reaction [2].

Next, we compared the performance of WIDOCK to that of the dedicated covalent docking algorithm of AD4. Contrarily to our protocol, we could not retrieve a significant amount of experimentally validated covalent inhibitors by the covalent docking available in AD4 within the custom classification threshold (Fig. 4a). Considering MurA, covalent docking showed a sensitivity of 20%, thus substantially lower than that achieved by WIDOCK (60%) (Fig. 4b). As for CatB, no actives were correctly predicted (sensitivity is equal to 0%). It is to be noted that the high sensitivity obtained by WIDOCK for CatB is accompanied with lower specificity owing to the generated false positives. This is in sharp contrast with the results obtained by covalent AD4 that generated a single false positive without identifying any true active within the custom threshold. Although the number of investigated compounds and experimental actives are clearly lower than it is typical in virtual screening campaigns, it is worth noting that the high sensitivity at the expense of modest specificity as found with WIDOCK allows the identification of actives although with increased experimental effort. The ROC curves (Fig. 4c) and the enrichment curves (Supporting Figure S4) clearly demonstrate the advantage that WIDOCK provides in terms of better TPR to FPR relation and high early enrichment of MurA and CatB actives as compared to covalent AD4, with the latter resulting in AUC values only slightly better than a random classification. It can also be seen how the lower specificity emphasized for WIDOCK against CatB is due to the incorrect positive classification of an additional 38% of the set following the identification of all true inhibitors (100% TPR already obtained in the top ranked 17% of the set).

**Fig. 5:**
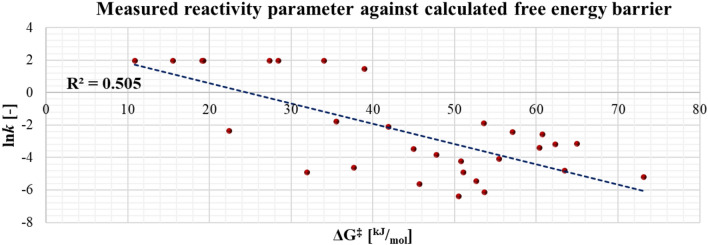
Correlation between calculated activation energy barriers (**ΔG**^**‡**^) and experimental reactivities (ln*k*)

We also investigated the ability of calculated reactivity descriptors (**ΔG**_***r***_, **ΔG**^**‡**^) for the parametrization of the pseudo-Lennard–Jones potentials. Reaction energy (**ΔG**_***r***_) did not turn out to be useful for this compound set with diverse warhead chemotypes (R^2^ = 0.066 between **ΔG**_***r***_ and the logarithm of the kinetic rate constant). Although the quantum chemical prediction of reactivity for diverse warheads is highly challenging, reasonable correlation was found (R^2^ = 0.505; RMSD = 1.99; N = 29) between **ΔG**^**‡**^ and the logarithm of the kinetic rate constant by considering all investigated warhead chemotypes (Fig. 5). The correlation was found to be statistically significant at the *p* = 1.08 × 10^–5^ level using a correlation t-test.

**Fig. 6:**
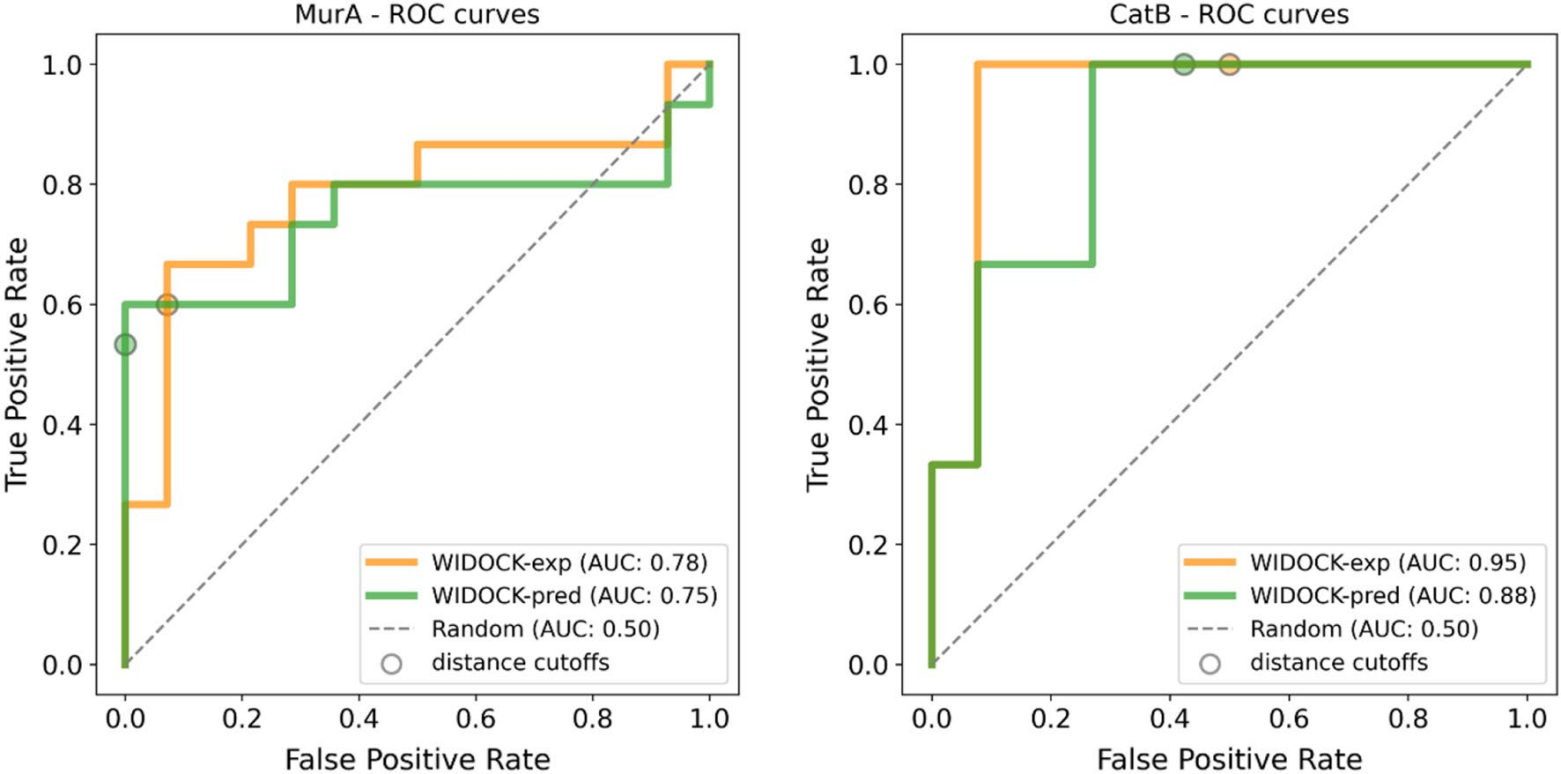
Comparison of ROC curves obtained using WIDOCK based on experimental (WIDOCK-exp, in orange) and calculated (WIDOCK-pred, in green) reactivity data against MurA and CatB

Therefore, we applied this model to predict kinetic rate constants for all compounds in the set, which were then used to derive the parameters of the pseudo-Lennard–Jones potentials for WIDOCK. The performance was shown to be highly similar to that obtained by the experimental reactivity parameters. In particular, considering the fraction of compounds within the distance cutoff, it resulted in 53% and 100% sensitivity against MurA and CatB, respectively (Fig. 6). Interestingly, equal (CatB) or similar (MurA) TPR values at distance cutoffs correspond to a higher specificity (1-FPR) for the protocol based on calculated parameters against both targets (100 and 58% for MurA and CatB, respectively).

**Fig. 7:**
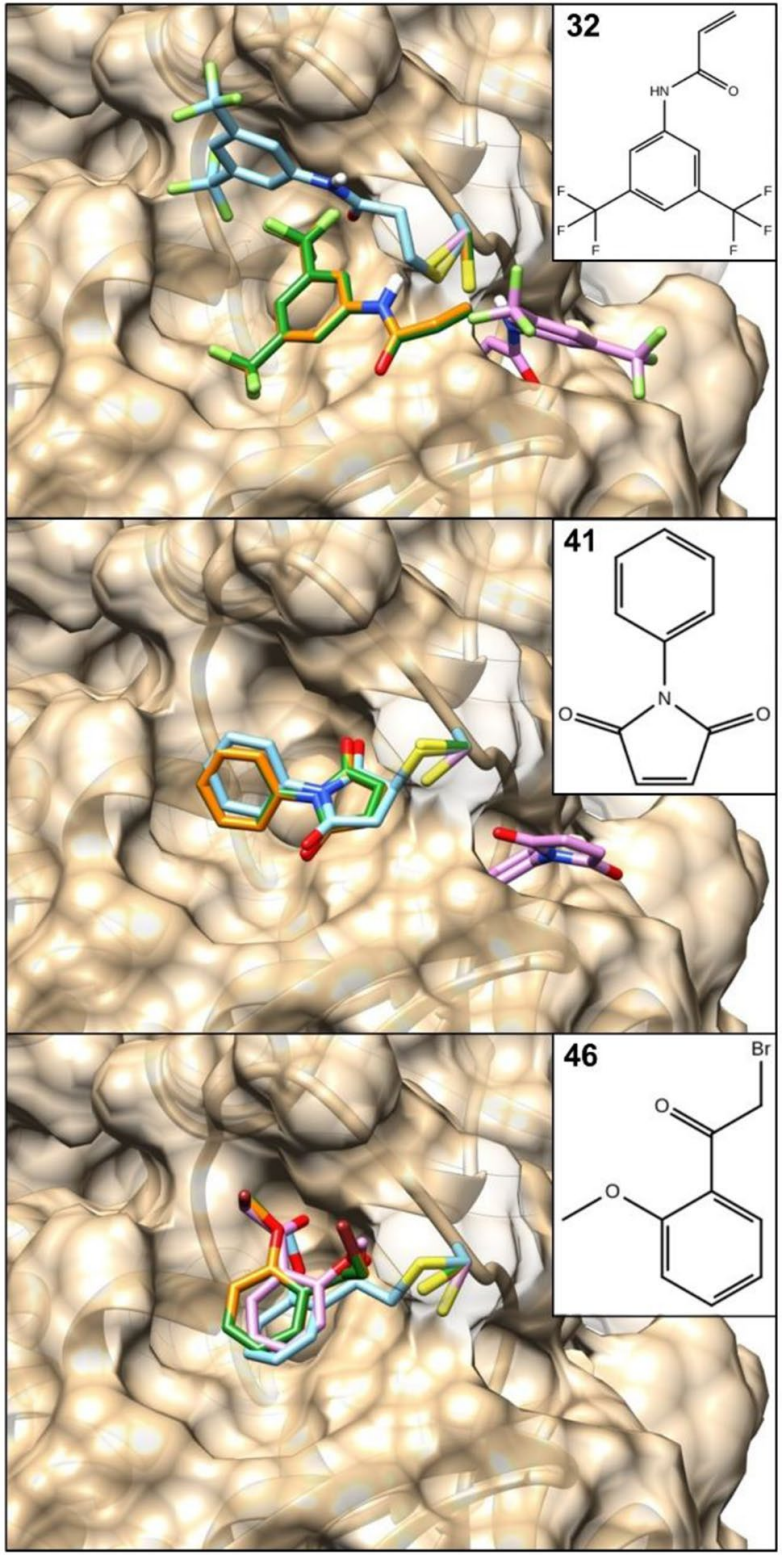
Surface representation showing the best scoring poses obtained by docking compounds **32**, **41** and **46** to MurA via different protocols. Standard non-covalent docking pose in pink; covalent docking pose in cyan; docking pose provided by WIDOCK parametrized with experimental reactivities in orange; docking pose provided by WIDOCK parametrized with predicted reactivities in green

We find it instructive to analyze docking poses obtained with WIDOCK and to compare them with the poses calculated by both non-covalent AD4 and covalent AD4. Figure 7 shows the conformations generated by all the evaluated methods when docking selected experimental MurA actives including maleimide (**41**), α-halo-acetophenone (**46**) and acrylamide (**32**) warheads. These three fragments were all predicted as actives by WIDOCK, while the covalent docking in AD4 only predicted the maleimide **41** among the best scored. While the methods provided different poses for **32**, the predicted binding modes of **41** and **46** by the covalent docking and WIDOCK overlapped significantly. In contrast, the standard non-covalent AD4 predicted fragments **32** and **41** into a different subpocket. Interestingly, an overlap is displayed by the phenyl ring in **46**, although in a flipped conformation that places the reactive carbon far from the reactive cysteine.

**Fig. 8:**
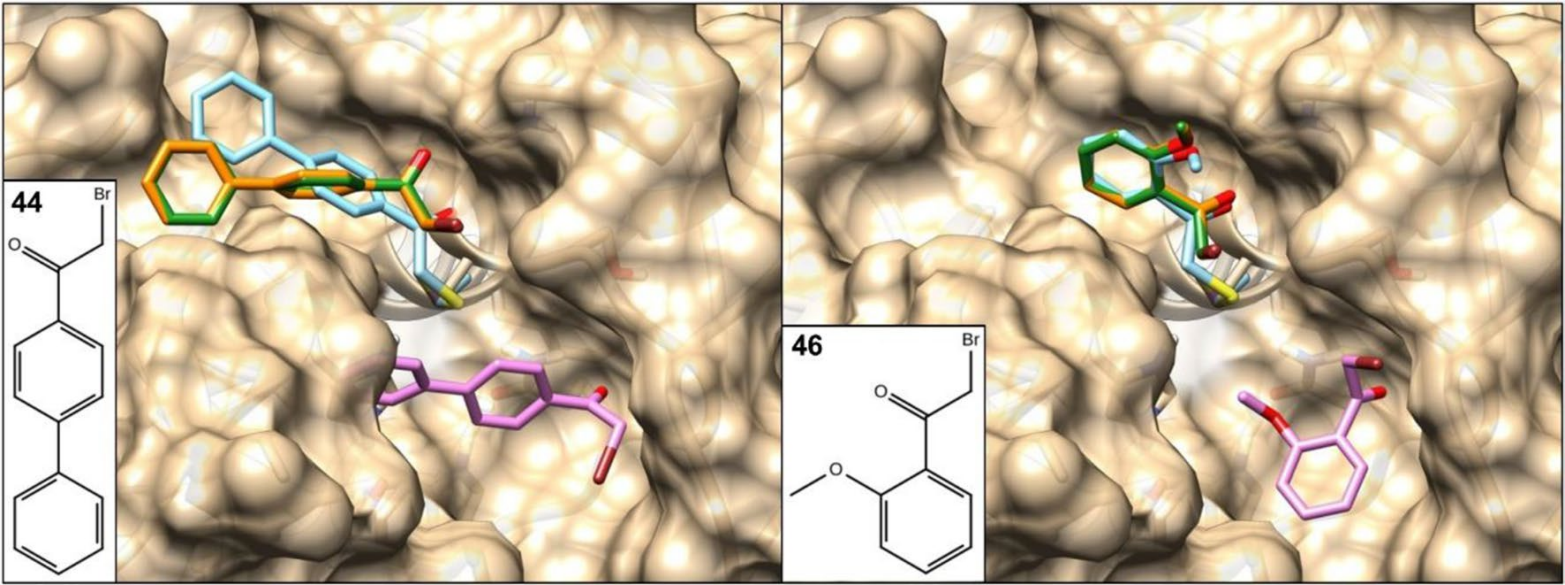
Surface representation showing the best scoring poses generated by docking compounds **44** and **46** against CatB via different protocols. Standard non-covalent docking pose in pink; covalent docking pose in cyan; docking pose provided by WIDOCK parametrized with experimental reactivities in orange; docking pose provided by WIDOCK parametrized with predicted reactivities in green

By docking our compound set to CatB, α-bromo-acetophenone was found as the only warhead type in experimentally validated inhibitors. WIDOCK was able to predict all the true actives in the set, although together with a higher number of false positives as compared to the other two targets. In Fig. 8, docking poses predicted for two experimental actives (**44** and **46**) are shown. As in the case of MurA, significant overlap was found between conformations generated by the covalent docking and WIDOCK, with only a slight deviation of the biphenyl system in **44** toward a different subpocket. In addition, both **44** and **46** clearly show the distinct binding mode that is produced by the non-covalent docking method neglecting the reactivity information.

**Fig. 9:**
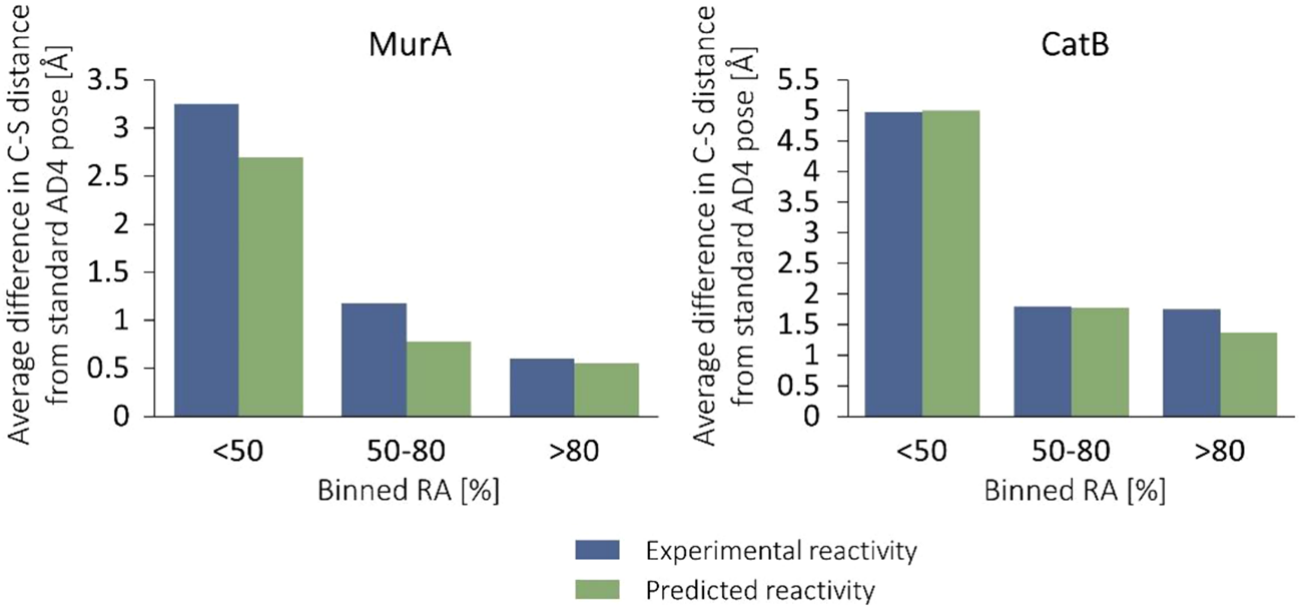
Differences of the distances between the reacting ligand atom and the cysteine sulfur as obtained with WIDOCK and with the non-covalent AD4 docking. Average differences plotted against binned remaining activities are shown

Interestingly, comparing the distances between the reactive atom pairs in the WIDOCK and non-covalent AD4 poses shows that their difference was increased parallel with the inhibitory activities (Fig. 9). The most striking differences involve those compounds that were predicted as covalent binders by WIDOCK, a significant fraction of whom were found as experimental actives. Altogether, these data clearly show that the improved sensitivity of WIDOCK can be traced back to the ligand reactivity considered within the docking process.

### Retrospective screening against OTUB2 and NUDT7

**Fig. 10:**
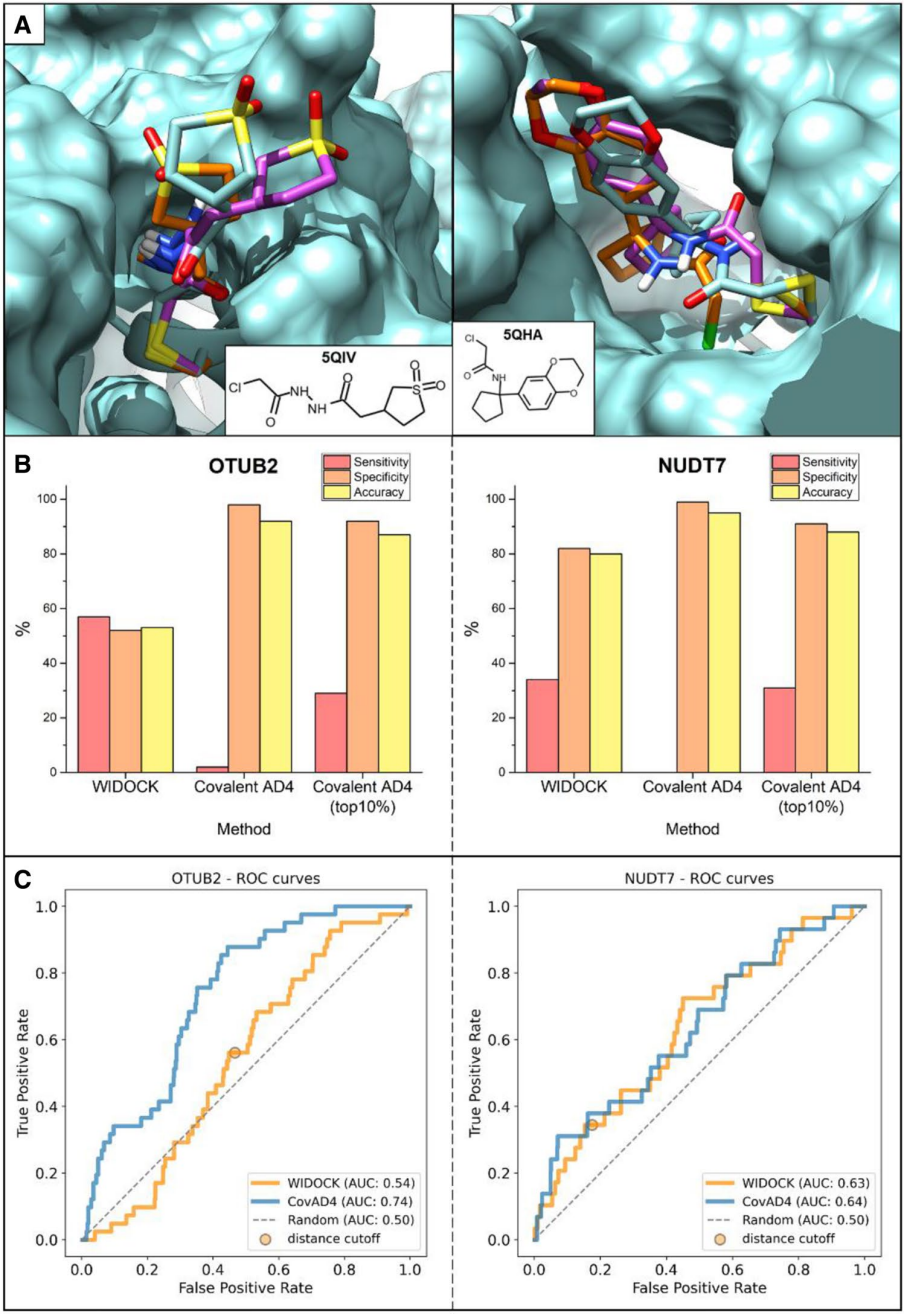
Evaluation of WIDOCK and covalent docking in AD4 against OTUB2 and NUDT7. **a** Docking poses for the compounds co-crystallized in the structures used for the virtual screening against OTUB2 (5QIV) and NUDT7 (5QHA). Crystal structure in cyan; covalent docking pose in purple; WIDOCK pose in orange. **b** Performance metrics at custom cutoffs. **c** ROC curves

To analyze the performance in a larger-scale virtual screening scenario, WIDOCK was applied on the set experimentally screened by Resnick et al. [43] against OTUB2 and NUDT7. The screening library consisted of mildly reactive chloroacetamides and acrylamides for which thiol reactivity data were reported, thus enabling the parametrization of WIDOCK. In detail, the logarithm of the average second-order kinetic rate constant (values in Supporting Table S5) was used to derive parameters for the pseudo-Lennard–Jones potentials, similarly to previously described applications. Overall, considering the performance at the usual distance cutoff, WIDOCK predicted 23 of the 41 experimental actives reported in the screening against OTUB2 (TPR = 56%) and 10 of the 29 actives found against NUDT7 (TPR = 34%). Structures of true positive hits for OTUB2 and NUDT7 are shown in Supporting Figure S3. Interestingly, all the true actives predicted by WIDOCK against NUDT7 (10/10) and most of those found against OTUB2 (20/23) were non-promiscuous hits in the screening carried out by Resnick et al. against ten different targets. The library was also screened by the covalent docking module in AD4 to have a direct comparison with a dedicated program. It is worth noting that such a large scale virtual screening application of covalent docking by AD4 is unprecedented and required automating the generation of post-reaction conformations. The evaluation of WIDOCK and covalent AD4 using the custom cutoffs and an extended cutoff of 10% for covalent AD4 is shown in Fig. 10b (for detailed results see Supporting Table S5). Evaluating the protocols using ROC curves (Fig. 10c), covalent AD4 showed a better performance compared to WIDOCK when applied against OTUB2. The dedicated covalent docking protocol could systematically enrich for more actives at all fractions of the screening set, while also predicting less false positives. This resulted in AUC values of 0.74 and 0.54 for covalent AD4 and WIDOCK, respectively. A different trend could instead be observed for the virtual screening against NUDT7, where the two docking protocols displayed highly comparable performances as shown by the respective ROC curves and enrichment plots (reported in Supporting Figure S4).

Docking poses predicted by WIDOCK and covalent docking for the compounds co-crystallized in 5QIV (OTUB2) and 5QHA (NUDT7) are shown in Fig. 10a. The targeted cysteines (Cys51 in OTUB2 and Cys73 in NUDT7) are located in pockets that are close to the surface, thus challenging the prediction of accurate conformations. The protocols could reproduce the overall shape and conformation of the experimental structures, although apparent deviations were found mainly in the solvent-exposed terminal of the ligands.

Overall, based on the results gathered on all retrospective studies, WIDOCK outperformed covalent AD4 when applied to libraries that included more diverse warhead chemotypes (KRAS^G12C^, MurA and CatB), while a comparable (NUDT7) or worse (OTUB2) performance was observed with compound sets spanning less variability in the reactive groups and thus in the intrinsic reactivities. Since WIDOCK was mainly devised to broaden the scope of electrophiles to be investigated in a virtual screening, these results confidently paved the way for its application in prospective studies to confirm its usefulness in the prediction of diverse covalent binders.

**Fig. 11:**
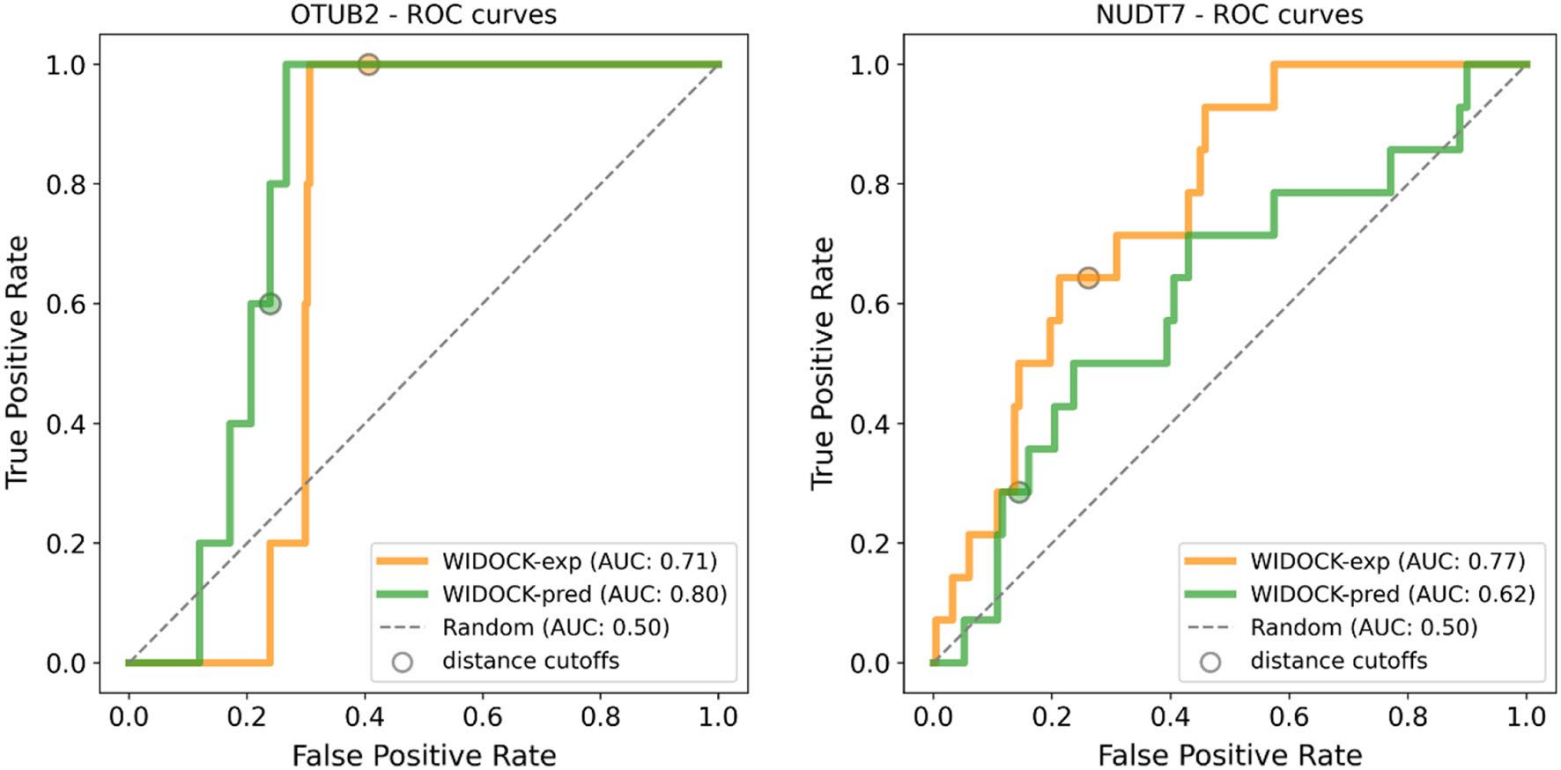
ROC curves for the virtual screening against OTUB2 and NUDT7 for a subset of compounds evaluated by WIDOCK with experimentally (WIDOCK-exp, in orange) and computationally derived (WIDOCK-pred, in green) reactivity parameters

Additionally, WIDOCK was tested against these two targets using the QM-based reactivity parameters described for the retrospective docking on MurA and CatB, in order to examine the applicability of the computational parametrization on larger screening sets. To ensure comparable reactivities, computationally derived parameters were assigned to compounds presenting a related warhead in the reference library screened versus MurA and CatB (for more details, see supplementary methods within the Supporting Information). By screening parametrized compounds against OTUB2, WIDOCK showed a sensitivity of 60%, a specificity of 76% and an overall accuracy of 76% considering compounds predicted within the defined distance cutoff. The screening results obtained for the same set of compounds but with experimentally derived parameters showed a higher sensitivity of 100% at the expense of reduced specificity (59%) and accuracy (60%). However, inspecting the performance via the respective enrichment and ROC curves, it is worth noting how the computational parametrization provided a better enrichment of actives in the initial third of the screening set and an overall higher AUC value compared to the protocol based on experimental parameters (Fig. 11 and Supporting Figure S4). When applied against NUDT7, the protocol with computational parametrization resulted in a sensitivity of 29%, with specificity and accuracy of 86 and 83%, respectively. Screening the same set of compounds with experimentally derived parameters exhibited 64% sensitivity and lower specificity (74%) and accuracy (73%) compared to the screening by computationally derived parameters. In general, WIDOCK with computationally derived parameters resulted in poorer performance for NUDT7, as the experimental parametrization could provide a consistently better enrichment of actives (Fig. 11 and Supporting Figure S4). It must also be emphasized that the experimental-based protocol predicted a larger fraction of compounds (~15%) within the distance cutoff against both OTUB2 and NUDT7, thus resulting in the identification of a higher number of actives compared to the computational protocol. However, these results confirmed that promising virtual screening hits can be predicted even by relying solely on a computational parametrization.

### Prospective screening against MAO-A by targeting an active site cysteine

Encouraging results obtained during the retrospective validation of WIDOCK prompted us to apply the protocol to identify new covalent MAO-A inhibitors. Although several known MAO-A inhibitors bind covalently to the FAD cofactor, to the best of our knowledge, no validated cysteine-binding covalent inhibitor has been reported yet for MAO-A. Inspecting the residues at the active site, we identified Cys323 in a position that its labelling is likely to block the access to the active site. Moreover, since two additional residues, Cys201 and Cys321, are found near the active site, the reactivity and accessibility of these three cysteines were characterized as explained in the previous section.

**Fig. 12:**
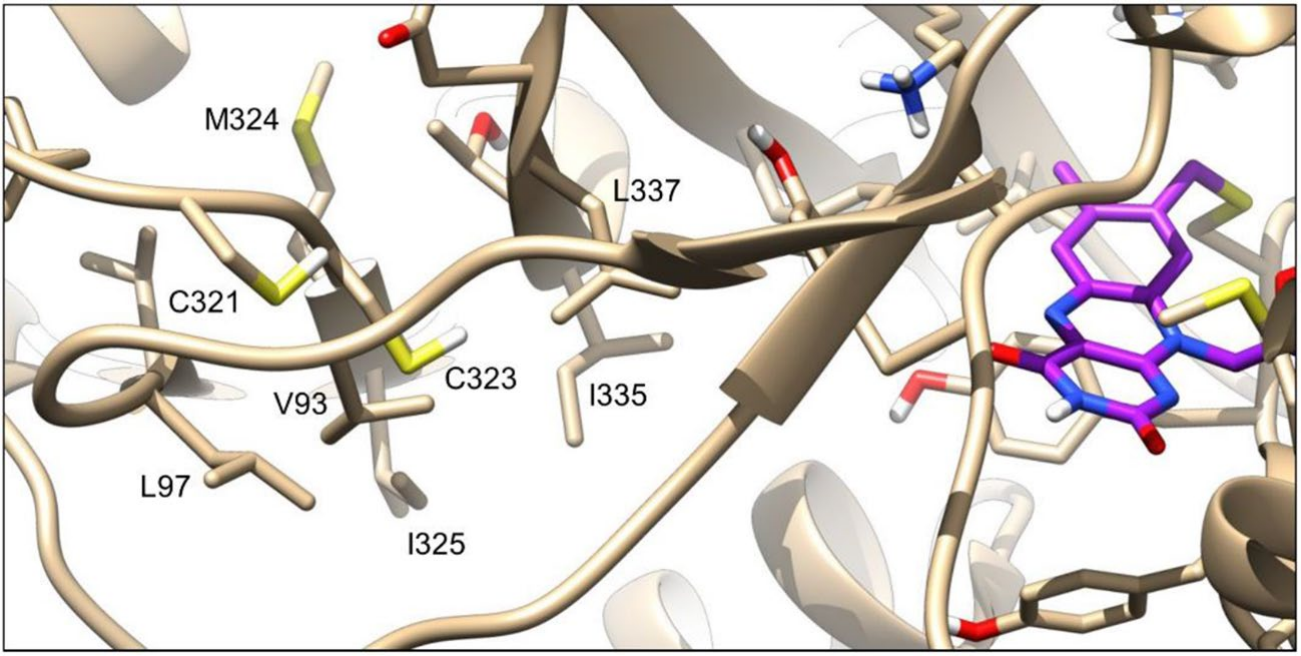
Active site of MAO-A with the FAD cofactor in purple, covalently bound to Cys406. Residues surrounding the active site Cys323 are shown

By comparing the values obtained for these three residues in MAO-A, we observed that Cys323 not only has the most negative ESP_min_, but also the largest SASA considering both the whole residue and the side chain sulfur (Table 3). These data suggest that Cys323 is the cysteine residue having the highest nucleophilic character and accessibility among the ones analyzed in MAO-A. Furthermore, Cpipe predicted it to be reactive, together with Cys321, despite their high pK_a_ values. It is worth noting that several cysteine residues for which labeling was proved by X-ray crystallography and/or MS experiments were found to have high predicted pK_a_ values [76]. Altogether, these data suggest that Cys323 is potentially targetable and support that Cys323 labeling may lead to MAO-A inhibition with a novel covalent mechanism of action. This hypothesis is supported by a recent report of several cysteine reactive covalent MAO-A inhibitors, however, their mechanism of action was not confirmed [77]. Figure 12 highlights the location of Cys323 and the surrounding residues (including Cys321) in the binding pocket of MAO-A.

**Table 3:** Parameters indicating reactivity and accessibility of cysteines in MAO-A

Target	Residue	QSite	POPS		Cpipe	
		ESP_min_ (kcal/mol)	Cys (whole) SASA (A^2^)	Cys (SG) SASA (A^2^)	pK_a_	Prediction
MAO-A	C201	− 170.41	8.17	2.25	11.23	Not reactive
	C321	− 285.77	10.18	3.99	10.12	Reactive
	C323	− 298.69	24.53	13.57	13.20	Reactive

**Fig. 13:**
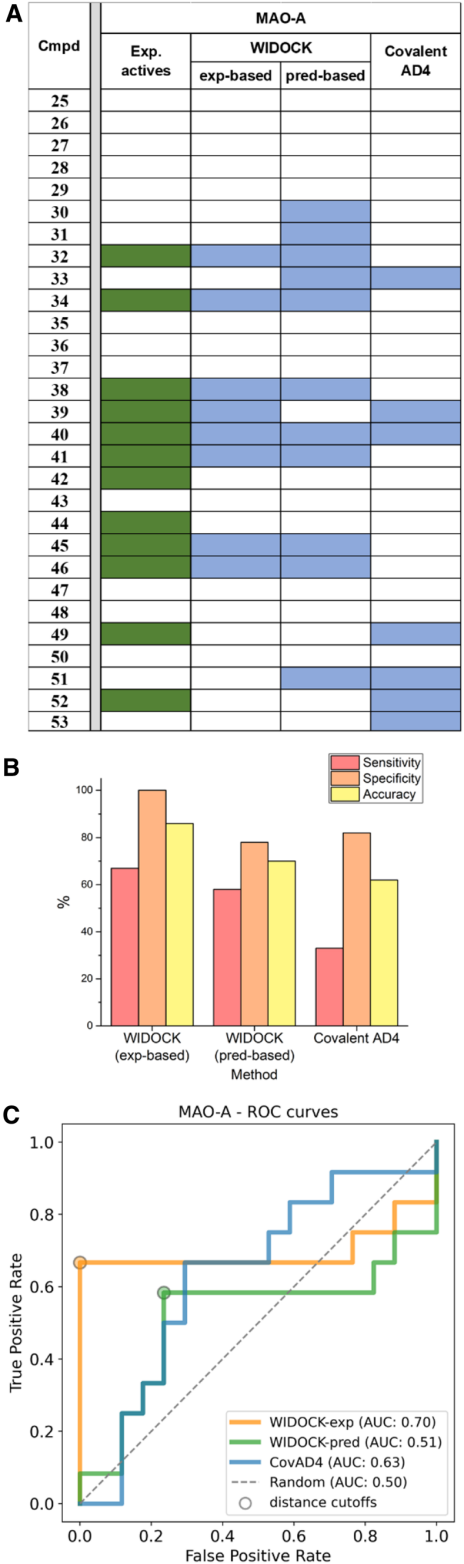
Evaluation of WIDOCK and covalent docking in AD4 against MAO-A. For WIDOCK, results obtained by using both experimental and predicted reactivity parameters are shown. **a** Docking results: colored cells represent experimental and predicted actives (in green and blue, respectively). **b** Performance metrics at custom cutoffs. **c** ROC curves

Compounds in Table 1 were docked with WIDOCK into MAO-A by using both experimental and computation-derived pseudo-Lennard–Jones potentials (see above). Virtual screening with standard covalent AD4 was also performed to analyze differences in the predictive power. All of the compounds were experimentally tested in MAO-A inhibitory assay. As summarized in Fig. 13a, applying WIDOCK with experimental reactivity parameters resulted in eight compounds predicted within the distance cutoff, which were all experimentally confirmed. Additional four compounds were found to inhibit MAO-A experimentally. These data represent 67% sensitivity, 100% specificity and overall 86% accuracy (Fig. 13b). The performance is slightly lower when the parameters of the potentials were derived from computed reactivities. In this case, one active is left unrecognized (compound **39**) and four false positives (**30**, **31**, **33**, **51**) appeared. Altogether, the sensitivity is 58%, the specificity is 76% and the accuracy is 69%. Thus, the hit rate with experimentally derived parameters is 100%, while it is 64% with the computationally derived parameters. Although the assessment of these results is affected by factors like the size and composition of the investigated library and the promiscuity of the identified inhibitors, the high hit rates are remarkable. Considering the performance of standard covalent AD4, the most notable difference is the lower sensitivity of 33%, as only four out of the 12 experimental actives were correctly predicted using the custom classification threshold, with a hit rate of 57%, accompanied by 82% specificity and 62% accuracy. By investigating the overall performance using ROC curves (Fig. 13c), the remarkably accurate classification ensured by WIDOCK based on experimental reactivity parameters is clearly shown by its ability to predict only active compounds within the specified distance cutoff, finally resulting in an AUC value of 0.70. On the other hand, the computational parametrization of WIDOCK provided a highly comparable performance relative to covalent AD4 within the first half of the library. Considering the fraction of compounds predicted within the distance cutoff by the purely computational protocol (top 38% of the set), covalent AD4 resulted in 50% sensitivity, 71% specificity and 62% accuracy, hence in a slightly poorer performance. Despite covalent AD4 had an overall larger AUC (0.63) compared to WIDOCK based on computational parameters (0.51), this is the result of a detrimental enrichment in the second half of the set past the distance cutoff (see also enrichment plots reported in Supporting Figure S4) and past also the region relevant in most virtual screening applications. Overall, the results collected in this prospective study confirmed a) the efficacy of WIDOCK in enriching for actives within the defined distance threshold, and b) its superior virtual screening performance compared to covalent AD4 when dealing with highly diverse warhead libraries.

The labelling of MAO-A by compound **32** was confirmed by MS/MS studies. Proteomics studies revealed that **32** forms a covalent bond with Cys323 located at the active site of MAO-A (Supporting Figure S5).

**Fig. 14:**
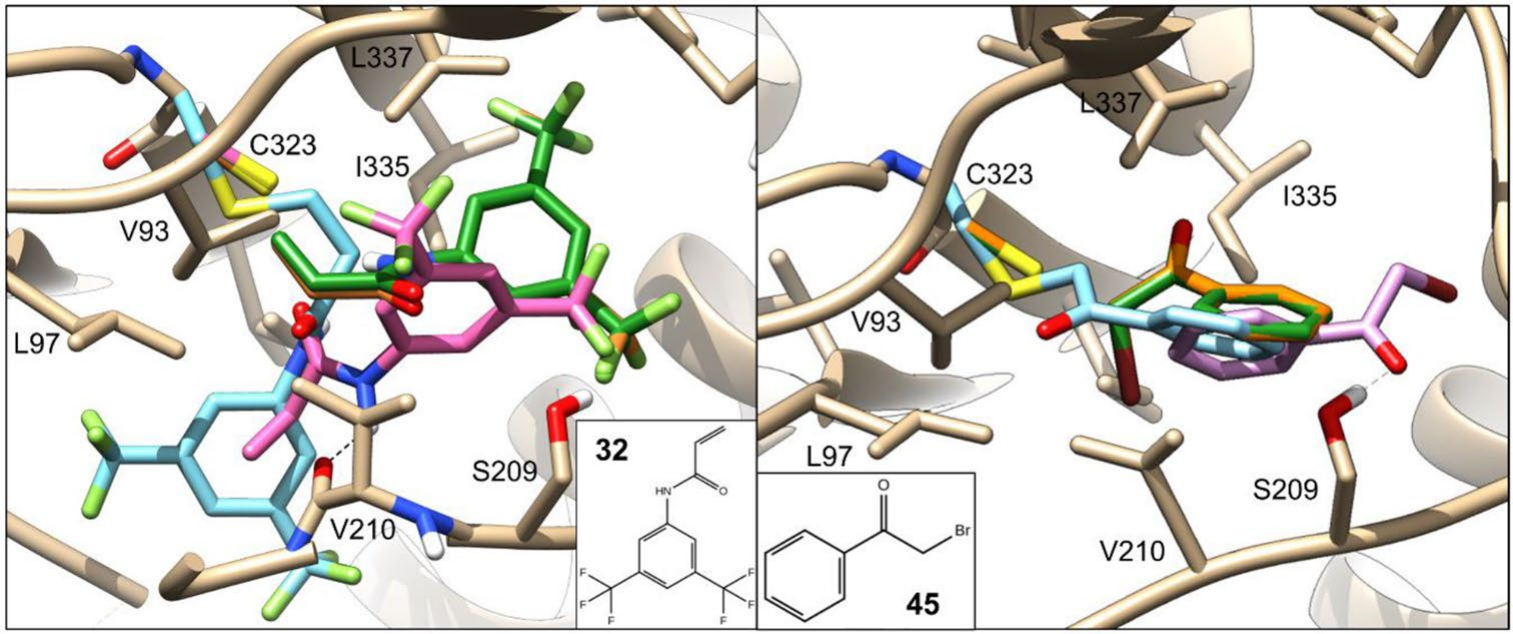
Best scoring conformations of compounds **32** and **45** against MAO-A obtained by different docking protocols. Non-covalent docking pose in pink; covalent docking pose in cyan; WIDOCK docking pose obtained by experimental and computed reactivity parameters in orange and green, respectively. Some residues were trimmed to aid the visualization

Docking poses predicted for two compounds found to inhibit MAO-A activity (**32** and **45**) are shown in Fig. 14. They illustrate how WIDOCK was able to predict active ligands bearing different warheads that react through different reaction mechanisms (**32** via Michael addition and **45** via nucleophilic substitution). Docking poses generated by the non-covalent docking in AD4 are used again as reference. For the acrylamide-based compound **32**, the reactive atom in the WIDOCK pose was found to be within bonding distance from the cysteine sulfur. By contrast, the best scoring pose provided by non-covalent docking placed the warhead farther away from the cysteine, showing a hydrogen bond interaction between the acrylamide-NH and the backbone carbonyl of Val210. The reactive carbon of the α-bromo-acetophenone **45** in the best scoring pose was placed at short distance from the cysteine sulfur by WIDOCK. On the other hand, non-covalent docking led to a flipped binding mode due to an H-bond interaction between the carbonyl oxygen and the hydroxyl group of Ser209. Covalent AD4 docking poses are also included to show differences in the predicted binding modes, although neither of the two was predicted among the best scoring ones by this protocol. Furthermore, poses generated by WIDOCK using experimental and computed reactivity parameters were found to be highly overlapping in the majority of cases, thus further supporting computational parametrization.

**Fig. 15:**
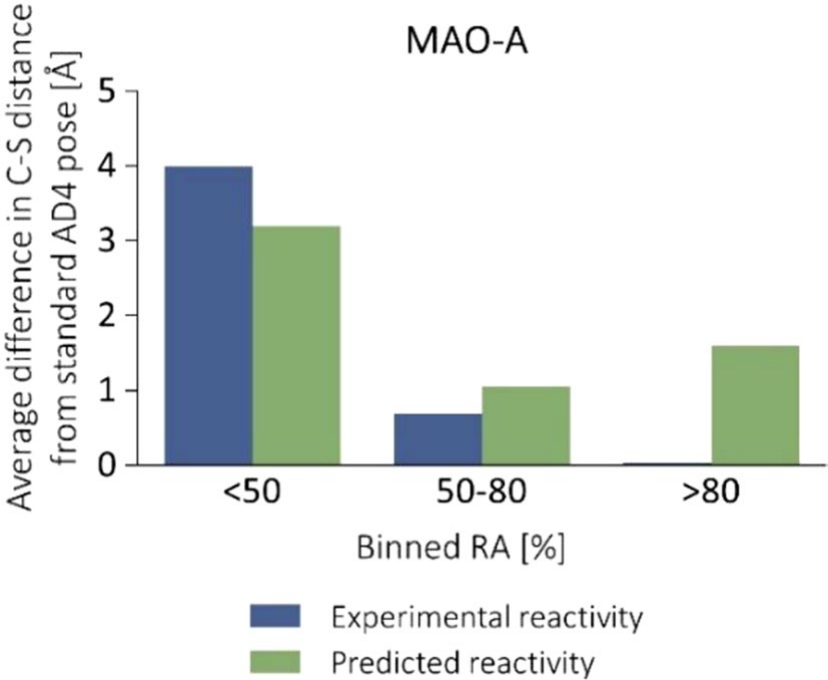
Differences of the distances between the reacting ligand atom and the cysteine sulfur as obtained with WIDOCK and with non-covalent AD4 docking. Average differences plotted against binned remaining activities are shown

We compared the distances between the reactive atom pairs in the poses provided by WIDOCK and by the standard non-covalent AD4. Similarly to what we observed for MurA and CatB (see Fig. 9), the difference increased parallel with the inhibitory activities (Fig. 15). These data confirm the tendency that the larger the inhibitory activity, the more pronounced the effect of the pseudo Lennard–Jones potential to produce short interatomic separation for the reacting atoms. This finding underlines the importance of including reactivity information in a docking protocol for covalent binders.

## Conclusion

Virtual screening of covalent inhibitors needs a robust docking-scoring scheme applicable to compounds with a wide range of covalent warheads. We presented WIDOCK as a reactive docking protocol that uses a ligand reactivity-based pseudo-Lennard–Jones potential in AutoDock4 to enable virtual screening of diverse warhead libraries. Ligand reactivities were derived from kinetic data obtained either from experiments or from quantum chemical calculations. WIDOCK was evaluated retrospectively against experimental data obtained for focused sets of diverse electrophiles against three targets, KRAS^G12C^, MurA and cathepsin B. Additionally, larger electrophilic fragment libraries with limited warhead diversity were screened against OTUB2 and NUDT7. Results were also contrasted to those obtained by covalent docking in AutoDock4. WIDOCK retrieved experimental actives with high sensitivity (true positive rate) and outperformed the dedicated covalent docking module of AutoDock4 in terms of early enrichments and ROC curves when screening libraries that spanned more diverse warhead chemotypes, while comparable or worse performances were obtained with sets characterized by lower variability in the reacting groups and in the corresponding intrinsic reactivities. When tested prospectively for discovering new MAO-A inhibitors with a new mechanism of action targeting Cys323, eight and seven actives (TPR: 67 and 58%) were identified with experimentally and computationally parametrized potentials. One of these compounds was proven to label Cys323 by subsequent MS proteomics measurements. To the best of our knowledge, this is the first experimentally validated case that MAO-A inhibition was achieved via direct Cys323 labelling. These results demonstrate that this warhead-sensitive docking protocol can be considered as a useful tool for the discovery of cysteine targeting covalent inhibitors. Furthermore, it was shown for compounds acting via Michael addition and nucleophilic substitution that the linear relationship between experimental and computed reactivities makes it possible to use computational parametrization of reactivity-based docking without a significant loss of accuracy. Therefore, we believe that the present parameter set of WIDOCK could be easily extended to new ligands acting with the same reaction mechanism. The warhead-sensitive nature of WIDOCK supports the parallel optimization of non-covalent and covalent interactions for the first time that might contribute to identify more specific and safer covalent inhibitors.

## Supplementary Information

Below is the link to the electronic supplementary material.

Supplementary file 1 (DOCX 1409 KB)

Supplementary file 2 (XLSX 37 KB)

Supplementary file 3 (XLSX 12 KB)

Supplementary file 4 (XLSX 16 KB)

Supplementary file 5 (XLSX 82 KB)

Supplementary file 6 (XLSX 18 KB)
