# Supplementary material for: WIDOCK: a reactive docking protocol for virtual screening of covalent inhibitors
Source: J Comput Aided Mol Des. 2021 Jan 18;35(2):223–44. doi: 10.1007/s10822-020-00371-5 (PMC7904743; doi:10.1007/s10822-020-00371-5)
Supplement: Supplementary file 1 — (DOCX 1409 KB) [file 10822_2020_371_MOESM1_ESM.docx]

**Supporting Information**

WIDOCK: a Reactive Docking Protocol for the Virtual Screening of Covalent Inhibitors

Andrea Scarpino^1^, László Petri^1^, Damijan Knez^2^, Tímea Imre^3^, Péter Ábrányi-Balogh^1^, György G. Ferenczy^1^, Stanislav Gobec^2^, György M. Keserű^1*^

^1^ Medicinal Chemistry Research Group, Research Centre for Natural Sciences, Hungarian Academy of Sciences, Magyar tudósok krt 2, H-1117 Budapest, Hungary

^2^ University of Ljubljana, Faculty of Pharmacy, Aškerčeva 7, SI-1000 Ljubljana, Slovenia

^3^ MS Metabolomic Research Laboratory, Research Centre for Natural Sciences, Magyar tudósok krt 2, H-1117 Budapest, Hungary

Corresponding Author

* György Miklós Keserű: keseru.gyorgy@ttk.hu

**
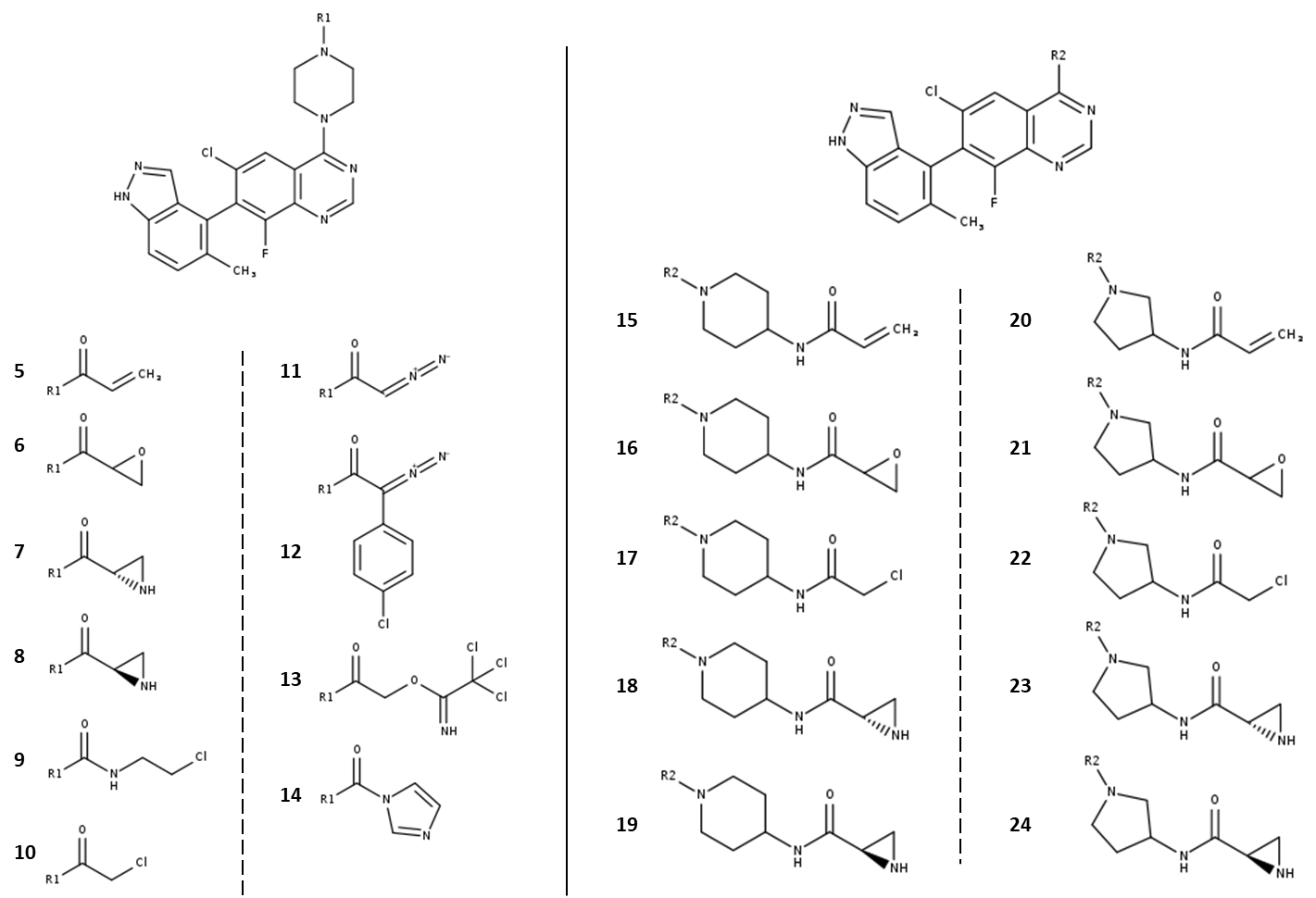
**

**Figure S1.** Compounds investigated against KRAS^G12C^.

**Figure S2.** Structures of fragments investigated against MurA, CatB and MAO-A.

**Table S1.** SMILES codes for evaluated compounds.

(provided in electronic format as SI_TableS1.xlsx)

**Table S2.** Molecular descriptors for fosfomycin, terreic acid and MurA actives.

| **Ligand(s)** | **All-atoms**  **Surface Area^(2)^** | **Labute’s Approximate Surface Area^(3)^** | **MW^(3)^** | **Heavy Atoms Count^(3)^** |
| --- | --- | --- | --- | --- |
| MurA actives**^(1)^** | 201.11 | 83.10 | 215.30 | 13.87 |
| Terreic acid | 146.20 | 62.46 | 154.03 | 11.00 |
| Fosfomycin | 130.66 | 46.63 | 138.01 | 8.00 |

^1^ all features are calculated as mean values across MurA actives

^2^ calculated with Schrödinger tools

^3^ calculated with RDKit

**Table S3.** Detailed results of the docking calculations against KRAS^G12C^: covalent AutoDock4, standard non-covalent AutoDock4 and WIDOCK applying experimental reactivity parameters.

(provided in electronic format as SI_TableS3.xlsx)

**Table S4.** Detailed results of the docking calculations: covalent AutoDock4, standard non-covalent AutoDock4 and WIDOCK applying experimental- and computational-based reactivity parameters. a) Results for MurA. b) Results for cathepsin B. c) Results for MAO-A. Experimental and predicted kinetic parameters are included.

(provided in electronic format as SI_TableS4.xlsx)

**Table S5.** Detailed results of the docking calculations against OTUB2 and NUDT7: covalent AutoDock4 and WIDOCK applying experimental and computational reactivity parameters.

(provided in electronic format as SI_TableS5.xlsx)

**Table S6.** Detailed results of the QM calculations to determine reaction energy (**ΔG*_r_***) and activation energy barrier (**ΔG^‡^**).

(provided in electronic format as SI_TableS6.xlsx)


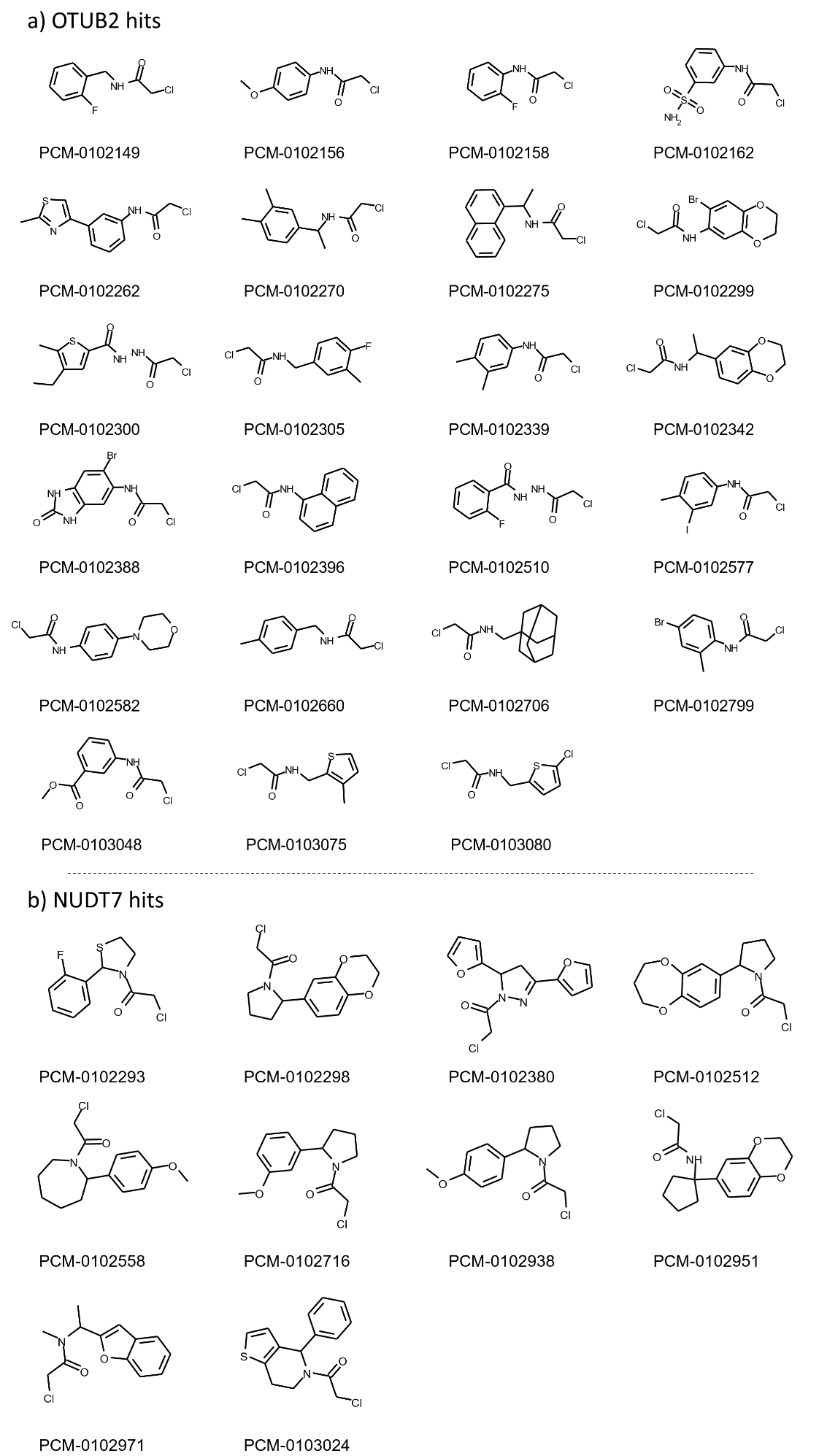


**Figure S3.** WIDOCK hits identified in the screening against OTUB2 (a) and NUDT7 (b). Compound names agree with those in the reference work (Resnick et al., *J. Am. Chem. Soc.* **2019**, *141*, 8951–8968). All compounds except OTUB2 hits PCM-0102158, PCM-0102262 and PCM-0102339 were found to be non-promiscuous in the referred screening campaign against 10 different targets.


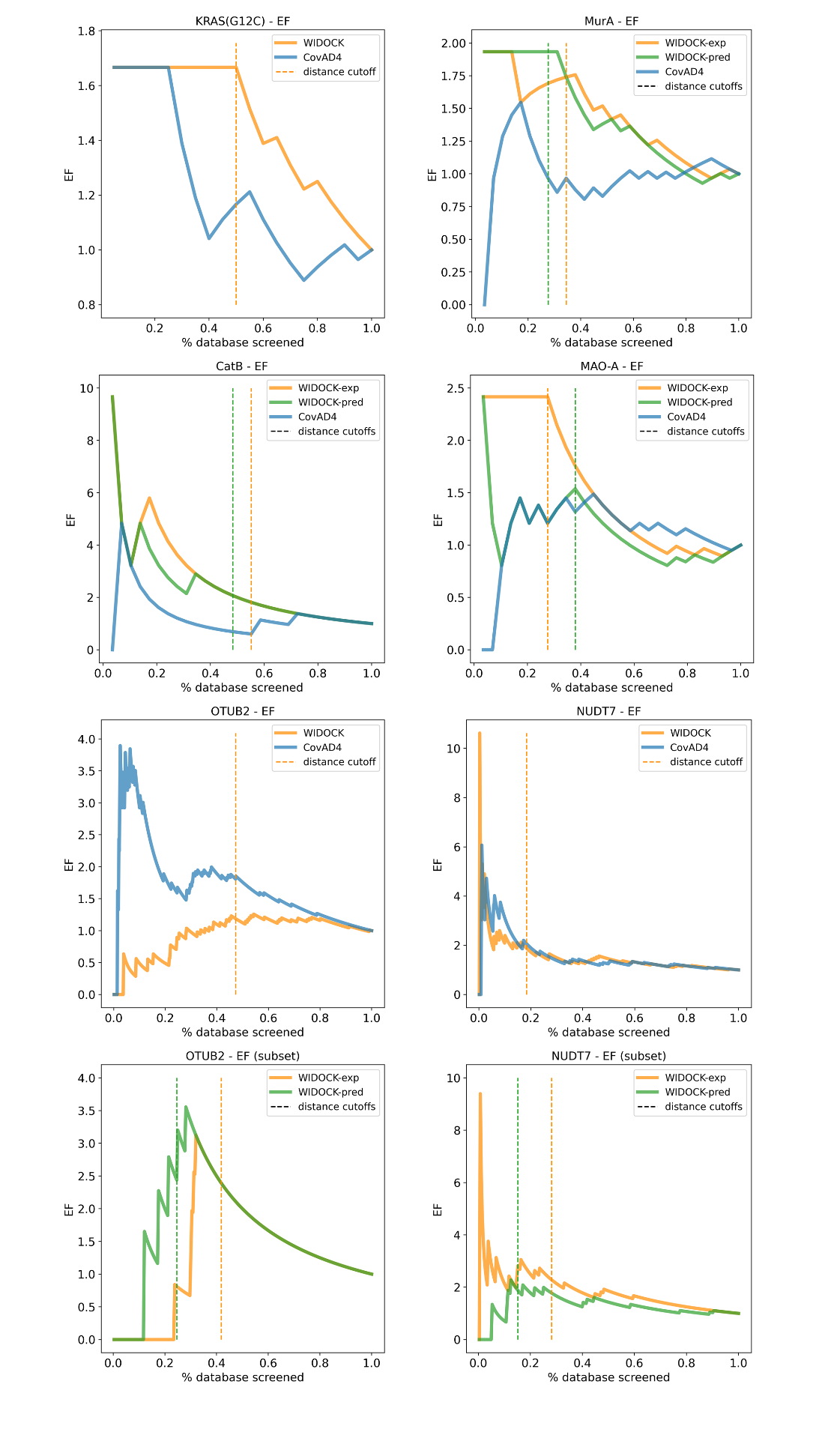


**Figure S4.** Enrichment plots for WIDOCK and covalent docking in AutoDock4 (CovAD4, in blue) in all case studies under investigation. For WIDOCK, plots are showed for the protocol using experimental reactivity parameters (in orange), unless stated otherwise. Plots obtained from the computational parametrization (WIDOCK-pred) are reported in green, where available.


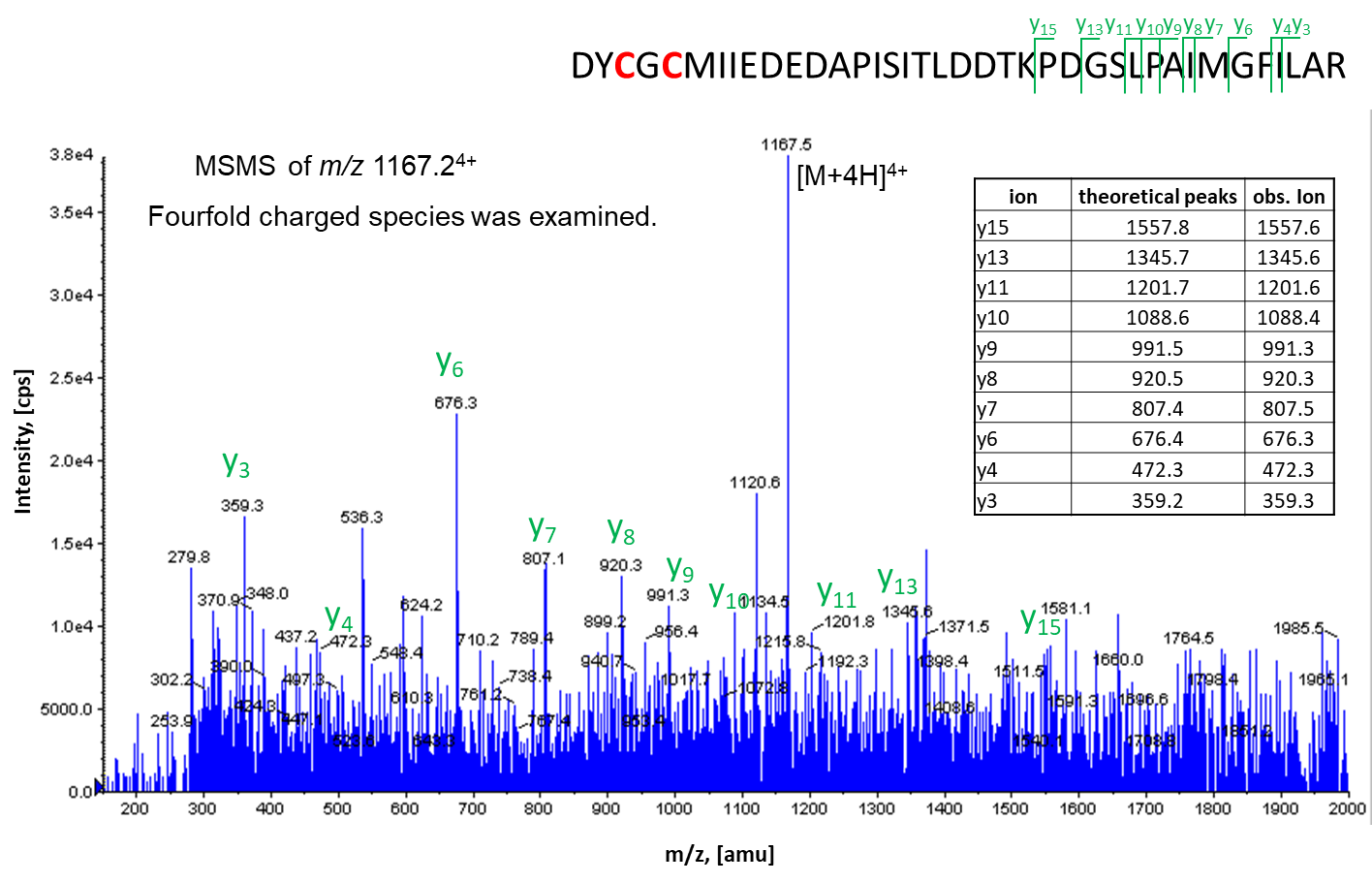


**Figure S5.** The MS/MS spectrum of **32** double modified MAO-A enzyme peptide fragment [319-356] together with the annotations.

**Supplementary methods**

1. ***Workflow description***

WIDOCK relies on the use of a pseudo-Lennard-Jones potential to model the interaction between reacting atoms on ligand and targeted cysteines. This potential is added to the default set of non-bonded interaction energies calculated during the docking simulation in AutoDock4. For this purpose, a new atom type must be defined in the default parameter file (AD4_parameters.dat) for the electrophilic carbon on the ligand and another one for the nucleophilic sulfur on the targeted cysteine residue. This is to ensure that the additional potential will not override pairwise energy evaluations involving other ligand-protein atoms.

A new line must be inserted in the parameter file for each of the new atom types, by keeping all the other parameters of the original ones. For instance, aliphatic carbon atoms are characterized by the following internal parameters in the default parameter file:

| atom type | Rii | epsii | vol | solpar | Rij_hb | epsij hb | hbond | rec index | map index | bond index |
| --- | --- | --- | --- | --- | --- | --- | --- | --- | --- | --- |
| C | 4.00 | 0.150 | 33.51 | -0.0014 | 0.0 | 0.0 | 0 | -1 | -1 | 0 |

To model the reactive β-carbon of an acrylamide warhead, a new line must be inserted in the parameter file by keeping the same values as the default aliphatic C, but with a different atom type name (e.g., CZ):

| atom type | Rii | epsii | vol | solpar | Rij_hb | epsij hb | hbond | rec index | map index | bond index |
| --- | --- | --- | --- | --- | --- | --- | --- | --- | --- | --- |
| CZ | 4.00 | 0.150 | 33.51 | -0.0014 | 0.0 | 0.0 | 0 | -1 | -1 | 0 |

Prior to generation of the grid maps (via prepare_gpf4.py script or AutoDockTools), the new atom types should replace the original ones in the ligand and protein .pdbqt files. This will produce a specific grid map file (*.CZ.map) for the electrophilic atom. In case of large compound libraries, this step can be automated by using a pattern recognition algorithm for the identification of the electrophilic atom to be modified in the .pdbqt file.

Then, a docking parameter file (*.dpf) is generated (via prepare_dpf4.py script or AutoDockTools). It should be modified by adding a new intnbp_r_eps keyword in order to instruct AutoDock to evaluate the custom pseudo-Lennard-Jones interaction potential between the specified atom types. The parameters for the new keyword are: r_eq_, ε, n, m, and the two atom types whose pairwise interaction should be assessed. In detail, r_eq_ is the equilibrium distance for the bottom of the potential, whose value was set to 1.80 Å being the optimal C-S bond length. The potential well depth (ε) must be set to the experimental/predicted thiol reactivity-scaled term. It must be noted that the parametrization of WIDOCK requires homogeneous reactivity data for the ligands in the screening set, as thiol reactivities resulting from different assays could not be directly compared. Therefore, each screening set in this study was investigated separately due to differences in the experimental assay. For each ligand in the set, ε was scaled linearly in a range between 0.175 and 1 according the corresponding kinetic parameter, following the reactive docking methodology. Finally, 13 and 7 were used as Lennard-Jones coefficients (n and m). For the calculation of Lennard-Jones potentials, AutoDock uses a simplified form obtained after rearrangement of the original equation. The new keyword will generate a potential according to the following equation:

$$V\left( r \right)\approx\frac{\frac{m}{n-m}\cdot\varepsilon\cdot r_{eq}^{n}}{r^{n}}-\frac{\frac{n}{n-m}\cdot\varepsilon\cdot r_{eq}^{m}}{r^{m}}$$

When evaluating the docking results (100 poses generated with default LGA settings), the best scoring pose is analysed. If the interatomic distance between the reacting atom pairs (C-S) is found to be within 2.20 Å (additional distance from the optimal bond length is allowed to account for van der Waals repulsions), then the compound is predicted to be a covalent binder.

1. ***Screening versus OTUB2 and NUDT7 using computational reactivity parameters***

Computational reactivity parameters calculated for the MurA/CatB set were used to parametrize pseudo-Lennard-Jones potentials for the compound libraries screened against OTUB2 and NUDT7. To reflect differences in reactivities, the vastly more represented chloroacetamides were filtered for compounds presenting comparable substitution patterns in the MurA/CatB set, and their parameters were mapped to the reference ones according to the following rules:

- NH-amide linked to *o*-substituted or *o*-,*p*-disubstituted phenyl ring (ref: 47)
- NH-amide linked to phenyl ring substituted in *m*- with e-withdrawing group (ref: 49)
- NH-amide linked to phenyl ring substituted in *m*- with e-donor group (ref: 51)
- *N*-,*N*-dialkylated amide (ref: 52)

This procedure led to two libraries of 256 (5 actives, 251 inactives) and 263 (14 actives, 249 inactives) ligands parametrized for virtual screening against OTUB2 and NUDT7, respectively. Compounds in the sets were assigned the same computationally derived pseudo-Lennard-Jones potential parameters as those of the corresponding reference ligands.
